# CPF-Associated Phosphatase Activity Opposes Condensin-Mediated Chromosome Condensation

**DOI:** 10.1371/journal.pgen.1004415

**Published:** 2014-06-19

**Authors:** Vincent Vanoosthuyse, Pénélope Legros, Sjaak J. A. van der Sar, Gaël Yvert, Kenji Toda, Thierry Le Bihan, Yoshinori Watanabe, Kevin Hardwick, Pascal Bernard

**Affiliations:** 1 CNRS, UMR5239, LBMC; Ecole Normale Supérieure de Lyon; Université Lyon 01, Lyon, France; 2 Wellcome Trust Centre for Cell Biology, University of Edinburgh, Edinburgh, United Kingdom; 3 Chromosome Dynamics, Institute of Molecular and Cellular Biosciences, The University of Tokyo, Yayoi, Bunkyo-ku, Tokyo, Japan; 4 SynthSys Edinburgh, The University of Edinburgh, Edinburgh, United Kingdom; EMBL Heidelberg, Germany

## Abstract

Functional links connecting gene transcription and condensin-mediated chromosome condensation have been established in species ranging from prokaryotes to vertebrates. However, the exact nature of these links remains misunderstood. Here we show in fission yeast that the 3′ end RNA processing factor Swd2.2, a component of the Cleavage and Polyadenylation Factor (CPF), is a negative regulator of condensin-mediated chromosome condensation. Lack of Swd2.2 does not affect the assembly of the CPF but reduces its association with chromatin. This causes only limited, context-dependent effects on gene expression and transcription termination. However, CPF-associated Swd2.2 is required for the association of Protein Phosphatase 1 PP1^Dis2^ with chromatin, through an interaction with Ppn1, a protein that we identify as the fission yeast homologue of vertebrate PNUTS. We demonstrate that Swd2.2, Ppn1 and PP1^Dis2^ form an independent module within the CPF, which provides an essential function in the absence of the CPF-associated Ssu72 phosphatase. We show that Ppn1 and Ssu72, like Swd2.2, are also negative regulators of condensin-mediated chromosome condensation. We conclude that Swd2.2 opposes condensin-mediated chromosome condensation by facilitating the function of the two CPF-associated phosphatases PP1 and Ssu72.

## Author Summary

Failure to properly condense chromosomes prior to their segregation in mitosis can lead to genome instability. The evolutionary-conserved condensin complex is key to the condensation process but the molecular mechanisms underlying its localization pattern on chromosomes remain unclear. Previous observations showed that the localization of condensin is intimately linked to regions of high transcription, although, somewhat paradoxically, its association with chromatin is disrupted by a processive polymerase activity. Here we identify several RNA processing factors as negative regulators of condensin in fission yeast. Two of these factors associate with PP1 phosphatase as an independent entity within the Cleavage and Polyadenylation Factor (CPF), a complex key for 3′ end RNA processing. Lack of this module induces only minor and context-dependent effects on gene expression. Our data suggest that this module helps maintaining the proper level of phosphatase activity within the CPF and thereby opposes the function of condensin in mitotic chromosome condensation.

## Introduction

Mitotic chromosome condensation is essential for genome integrity. When defective, chromatin bridges often form in anaphase. These can lead to chromosome breaks and the irreparable loss of genetic information. A key driver of chromosome condensation is the highly conserved condensin complex (reviewed in [1]). Condensin is, with cohesin and the SMC5/6 complex, one of three highly conserved multi-subunit protein complexes containing two different proteins of the SMC (Structural Maintenance of Chromosome) family. Condensin is made of five sub-units (SMC2^Cut14^, SMC4^Cut3^, CAP-D2^Cnd1^, CAP-G^Cnd3^ and CAP-H^Cnd2^, name of the human protein followed by its name in fission yeast), which together form a protein ring big enough to entrap two chromatids. Condensin exhibits a DNA-dependent ATPase activity and a DNA supercoiling activity but how these enzymatic activities contribute to mitotic condensation remains elusive. Although condensin interacts directly with histones [2], its localization pattern along chromosomes is not uniform [3], [4]. A number of experimental evidence indicate that *cis*-acting elements facilitate the binding of condensin at specific loci, supporting the current view that the underlying mechanisms of condensin recruitment are, to some extent, locus-specific (reviewed in [1]).

However, Chromatin Immunoprecipitation (ChIP) studies also indicate that condensin localizes preferentially at highly expressed genes, irrespective of the transcription machinery involved (RNA Pol I, II or III) [3], [4], [5]. This observation supports the idea that a by-product of the transcription process, such as a transcription-associated chromatin structure, a specific change of topology and/or a chromatin mark, could facilitate the recruitment of condensin. It is likely that the recruitment of condensin actually results from the combination of global and locus-specific mechanisms. Our understanding of these mechanisms and their interactions remains poor.

Experimental evidence indicates that there are strong functional relationships between gene transcription and mitotic chromosome condensation. However some observations suggest that the transcription machinery plays a positive role in condensin-mediated chromosome condensation whilst other evidence indicates that gene transcription can inhibit chromosome condensation. On one hand, specific transcription-associated factors and RNA processing factors, such as the RNA Pol III component TFIIIC and the RNA helicase DDX3, have been shown to facilitate the loading of condensin [3], [6], [7],[8]; on the other hand, the stable association of RNA with chromatin maintains an open chromatin conformation [9] and an active RNA polymerase reduces the binding of condensin at repetitive sequences [10], [11]. Moreover, the transcription machinery can recruit inhibitors of condensin such as the phosphatase PP2A [12]. Finally, transcription of Pol III genes negatively correlates with their condensin-dependent clustering at centromeres [6]. In conclusion, it appears that, although the transcription machinery is required to set up the right environment for the loading of condensin, transcription, when processive, destabilizes the association of condensin with chromatin. Interestingly, a number of proteins required for the processivity of transcription leave chromatin upon mitotic entry, when condensin is loaded on chromosomes and condensation occurs [13], [14].

To gain insights into the mechanisms by which transcription influences the association of condensin with chromatin in fission yeast, we set out to identify novel regulators of chromosome condensation associated with the transcription machinery. To do this, we adopted a candidate approach and screened for deletions of non-essential components of the transcription machinery that could restore cell growth of the thermo-sensitive and condensin-defective *cut3-477* mutant at high temperatures. We identified a number of suppressor mutations by this approach. Here we focus on one of them, *swd2.2Δ*, the deletion of a non-essential component of the Cleavage and Polyadenylation Factor (CPF), the complex responsible for the 3′end maturation of RNA Pol II transcripts in yeast (reviewed in [15]).

## Results

### Lack of Swd2.2 restores chromosome segregation in a condensin-deficient mutant

**Figure 1:**
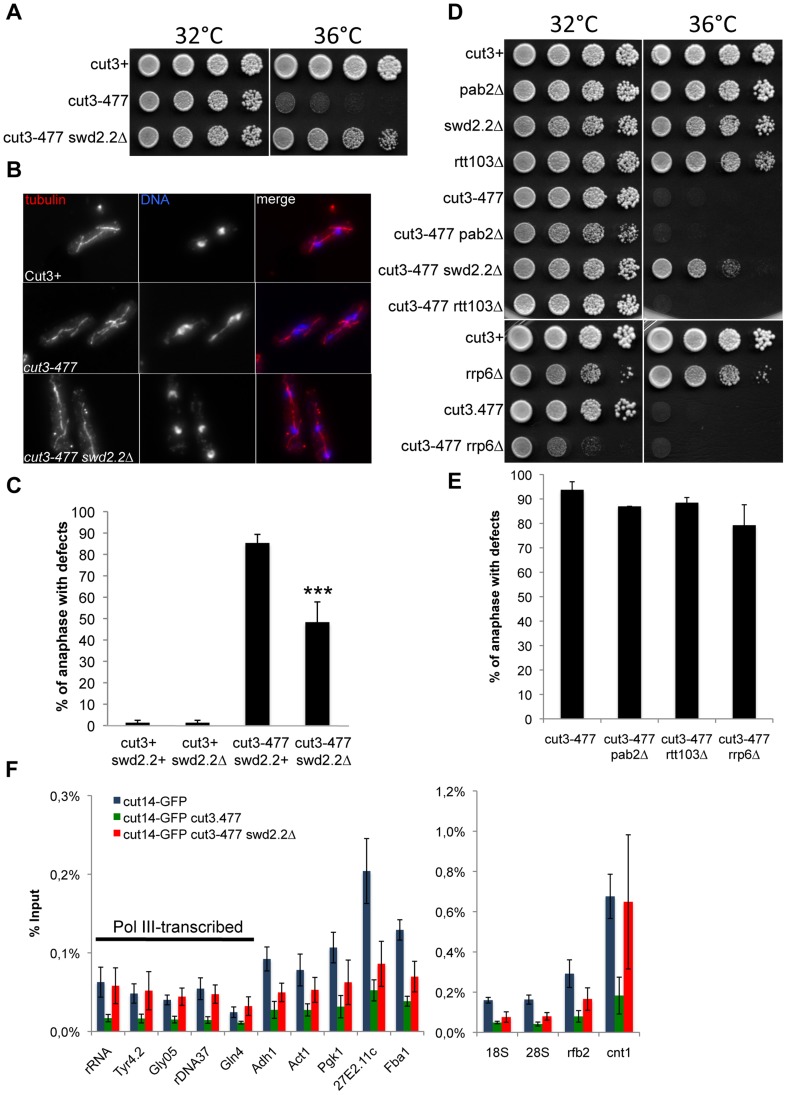
Swd2.2 antagonizes the association of Condensin with chromatin. **A.** Serial dilutions of the indicated strains were plated on rich media at the indicated temperatures. **B.** Chromosome segregation in anaphase was visualized after tubulin staining in cells of the indicated genotypes grown for one generation at 34°C. **C**. Anaphases were scored as defective when chromatin was detected lagging between the two main DNA masses. For each genotype, a minimum of 6 independent experiments was performed in which a minimum of 100 anaphase cells was scored. ***p<0,001 Wilcoxon - Mann Whitney. **D** and **E**. Same as **A** and **C**, except that in **E**, 3 independent experiments were performed. **F.** The indicated strains were grown at 34°C for 3 hours and ChIP-qPCR was performed to analyze the amount of Cut14-GFP cross-linked to chromatin (mean ± standard deviation from 6 biological replicates). See text for details for the statistical analysis of the experiments.

Chromosome condensation is defective in the conditional condensin mutant *cut3-477* and mutant cells fail to grow at the restrictive temperature of 34°C [16]. We isolated several gene deletions in non-essential components of the transcription machinery which partly restored growth of *cut3-477* cells at 34°C (Table S1). Here, we focused on one of the strongest of these suppressors, *swd2.2Δ*. At the restrictive temperature, lack of Swd2.2 (*swd2.2Δ*) improved the growth of *cut3-477* cells (Figure 1A) and significantly decreased the percentage of anaphases displaying defective chromosome segregation (Figure 1BC). Similarly, lack of Swd2.2 partly restored growth at the restrictive temperature of the other well-characterized condensin mutant, *cut14-208* [16] (Figure S1A). This suggested that Swd2.2 interferes with the formation of segregation-competent chromosomes when condensin is deficient. Alternatively, lack of Swd2.2 could activate an as yet unknown mechanism facilitating chromosome segregation in condensin-deficient cells.

The suppression of *cut3-477* by *swd2.2Δ* was remarkably specific. Deletions of other key components of the RNA processing machinery did not rescue either the growth defect or the chromosome segregation defects in *cut3-477* cells (Figure 1DE), indicating that significant RNA processing defects are not sufficient to alleviate the defects caused by *cut3-477*. For example, lack of either Rtt103/Rhn1 [17], [18], a factor important for transcription termination, Pab2, a PolyA polymerase involved in RNA decay or the exosome sub-unit Rrp6 [19] caused no significant rescue of *cut3-477* (Figure 1DE). Furthermore, lack of Swd2.2 did not rescue the *rad21-K1* and *smc6-74* mutations (Figure S1B), which affect respectively the condensin-related cohesin and Smc5/6 complexes, nor the two topoisomerase II mutations, *top2-250* and *top2-191* (Figure S1B), which also cause chromosome condensation defects [20], [21]. These observations support the idea that Swd2.2 specifically antagonizes condensin-mediated chromosome condensation.

### Lack of Swd2.2 facilitates the localization of condensin in *cut3-477* cells

It was reported that the amount of condensin associated with chromatin is reduced in *cut3-477* cells [2]. We confirmed these observations by showing that the association with chromatin of the GFP-tagged condensin sub-unit Cut14 was drastically reduced in *cut3-477* cells at the restrictive temperature of 34°C at all loci tested (p<0,01 Mann-Whitney test on 6 biological replicates, Figure 1F). Deletion of Swd2.2 improved slightly but significantly the association of Cut14 with chromatin in *cut3-477* cells at 34°C (p≤0,01 Mann-Whitney test on 6 biological replicates). Surprisingly, we found that the localization of Cut14 was fully restored at kinetochores (cnt1) and RNA Pol III-transcribed genes, where its enrichment was indistinguishable in cut3+ and *cut3-477 swd2.2Δ* cells (p>0,30 Mann-Whitney test). Western blot analysis showed that the steady-state level of Cut14 was not affected in cells lacking Swd2.2 (Figure S2). Consistent with the observation that *swd2.2Δ* facilitates the localization of condensin at kinetochores in *cut3-477* cells, Figure S3 shows that lack of Swd2.2 suppressed the synthetic lethal interaction between cut3-477 and *mde4Δ*, a cis-acting loader of condensin at kinetochores ([2]).

Taken together, these data suggest that Swd2.2 antagonizes the association of condensin with chromatin in *cut3-477* cells. This probably explains why lack of Swd2.2 reduced the temperature-sensitivity of *cut3-477*. Note however that, in cut3+ cells, lack of Swd2.2 did not significantly affect the association of condensin with chromatin (Figure S4).

### Lack of Swd2.2 did not alter Cnd2 phosphorylation or H2A.z acetylation

Previous experiments have established that the interaction between condensin and chromatin is enhanced after Aurora B-dependent phosphorylation of Cnd2 [2] and that the acetylation of H2A.z facilitates the function of condensin [22]. We wondered whether Swd2.2 might counter-act Aurora B-dependent phosphorylation of Cnd2 or H2A.z acetylation. Western blot analysis showed that neither Aurora B-dependent Cnd2 phosphorylation nor H2A.z acetylation were altered in the absence of Swd2.2 (Figure S5), suggesting that Swd2.2 acts upon condensin binding by an alternative pathway.

### Lack of Swd2.2 impairs the association of the Pol III machinery with chromatin

**Figure 2:**
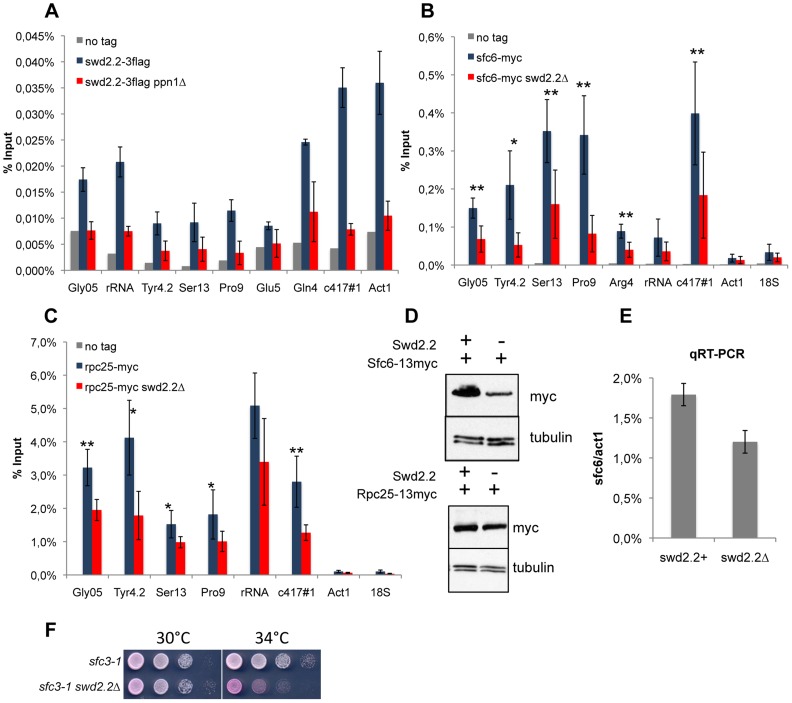
Lack of Swd2.2 reduces the association of the RNA Pol III transcription machinery with chromatin. **A.** Asynchronous populations of the indicated strains were grown at 30°C and ChIP-qPCR was performed to analyze the amount of Swd2.2-3flag cross-linked to chromatin (mean ± standard deviation from 4 biological replicates). **BC.** ChIP qPCR analysis of the indicated strains grown at 30°C. Mean ± standard deviation from 5 biological replicates. *<0,05; **<0,01; Wilcoxon - Mann Whitney. **D.** Western blot analysis of total protein extracts of the indicated strains. Tubulin (TAT1 antibody) is used as a loading control. **E.** qRT-PCR analysis of sfc6 expression in swd2.2+ and *swd2.2Δ* cells (mean ± standard deviation from 3 biological replicates) **F.** Serial dilutions of the indicated strains were plated on rich media at the indicated temperatures.

We asked how Swd2.2 could regulate the localization of condensin at Pol III-transcribed genes in *cut3-477* cells. We detected variable amounts of Swd2.2 at Pol III-transcribed loci by ChIP analysis. At some Pol III genes such as *c417* or *Gln4*, the enrichment of Swd2.2 was comparable to its enrichment at highly transcribed Pol II genes such as *act1* (Figure 2A). At all Pol III loci, the association of Swd2.2 was dependent on the PNUTS homologue Ppn1 (Figure 2A and see below). The localization of Swd2.2 at Pol III genes suggested that it could play a direct role there.

Observations made previously in the fission yeast TFIIIC mutant *sfc3-1* [6], suggested that TFIIIC binds to condensin and facilitates its association with Pol III genes, whereas Pol III-dependent transcription opposes the function of condensin. *sfc3-1*, like *swd2.2Δ*, is a suppressor of *cut3-477* and is believed to facilitate the localization of condensin at Pol III loci [2], [6]. Previous reports indicated that the association of the TFIIIC component Sfc6-13myc with chromatin was stabilized in *sfc3-1* cells, whilst the binding of the RNA Pol III sub-unit Rpc25 was reduced [6]. On the contrary, we found that both the association of Sfc6-13myc and Rpc25-13myc were reduced in *swd2.2Δ* cells (Figure 2BC). The reduced association of Sfc6-13myc with chromatin could probably be explained in part by the fact that both gene expression and the protein stability of Sfc6 were reduced in *swd2.2Δ* cells (Figure 2DE). The stability of Rpc25-13myc however remained unaffected (Figure 2D). As a control, we repeated the Sfc6-13myc ChIP in *sfc3-1* cells and found the opposite result to what is published [6]: in our hands, the localization of Sfc6-13myc was significantly impaired in *sfc3-1* cells (Figure S6B). We confirmed however the published observation [6] that the localization of Rpc25 is indeed slightly reduced in *sfc3-1* cells (Figure S6C). All our strains have been thoroughly validated (Figures S6A). We do not have an explanation for this discrepancy at this stage. We note however that lack of Swd2.2 strongly impaired the growth of *sfc3-1* cells (Figure 2F), which is consistent with our observation that both *swd2.2Δ* and *sfc3-1* destabilize the loading of TFIIIC. A common feature of both types of suppressors (*sfc3-1* and *swd2.2Δ*) is therefore that the loading of TFIIIC and RNA Pol III is impaired at Pol III genes.

### Swd2.2 facilitates the localization of the CPF but does not impact its assembly

**Figure 3:**
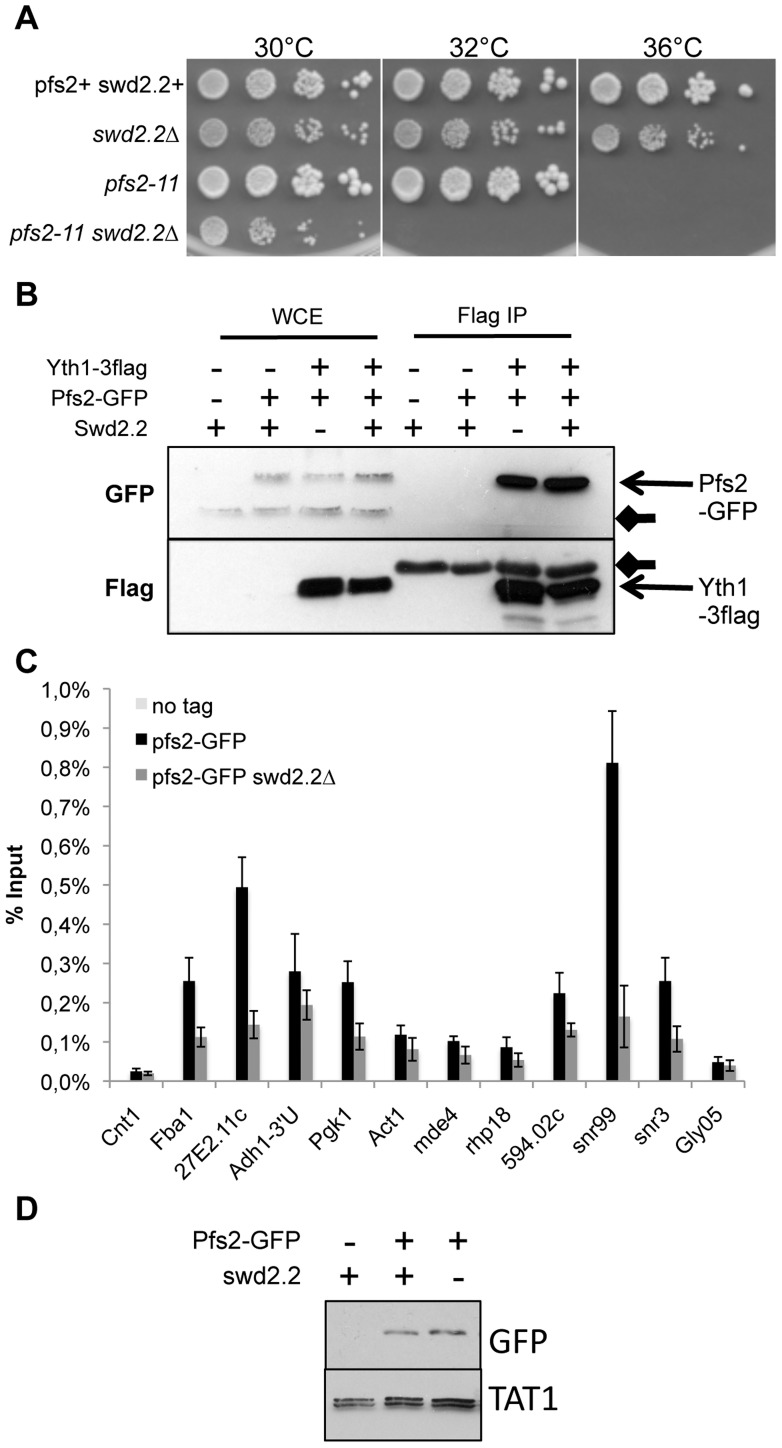
Swd2.2 facilitates the function of the CPF. **A.** Serial dilutions of the indicated strains were plated on rich media at the indicated temperatures. **B.** The CPF component Yth1 tagged at the endogenous locus with 3flag epitopes was immuno-precipitated from cycling cells in the presence or absence of Swd2.2. Whole cell extracts (WCE) and the immuno-precipitated material (Flag IP) were analyzed by western blot. Yth1-3flag interacts with Pfs2-GFP whether or not Swd2.2 is present. Arrows indicate aspecific bands on the western blot. **C.** Asynchronous populations of the indicated strains were grown at 30°C and ChIP-qPCR was performed to analyze the amount of Pfs2-GFP cross-linked to chromatin (mean ± standard deviation from 4 biological replicates). **D.** Western blot analysis of total protein extracts of the indicated strains. Tubulin (TAT1 antibody) is used as a loading control.

Swd2.2 was previously shown to co-purify with the Cleavage and Polyadenylation Factor (CPF) [23], the complex responsible for the 3′end maturation of RNA Pol II transcripts in yeast (reviewed in [15]). We sought to establish the importance of Swd2.2 for CPF function. *pfs2-11* is a thermo-sensitive mutation of Pfs2, an essential component of the CPF [24]. Lack of Swd2.2 enhanced the temperature-sensitivity of *pfs2-11* (Figure 3A), which is consistent with the idea that Swd2.2 could facilitate CPF function. However, lack of Swd2.2 did not interfere with the interaction between Pfs2 and Yth1, another essential CPF sub-units, suggesting that Swd2.2 did not significantly impact CPF assembly (Figure 3B and see below). To establish whether lack of Swd2.2 could impact CPF localization, we analyzed the association of Pfs2 with chromatin by Chromatin Immunoprecipitation (ChIP). The enrichment of Pfs2 was mildly reduced at most loci tested in the absence of Swd2.2, suggesting that Swd2.2 facilitates the recruitment of the CPF (Figure 3C). Note that the stability of Pfs2 remained unaffected in the absence of Swd2.2 (Figure 3D).

### Lack of Swd2.2 has a minor effect on the expression of protein-coding genes

To establish the impact of Swd2.2 on gene expression, we used strand-specific tiling arrays hybridization to compare the transcriptomes of *swd2.2Δ* and swd2.2+ strains (raw data accessible at GEO GSE38005). Both strains were grown in triplicates for one generation at 34°C, the temperature at which lack of Swd2.2 improves chromosome segregation in *cut3-477* (Figure 1A). Results showed that 47 genes were significantly under-expressed in the absence of Swd2.2 (Fold change FC≤−1,9, p<0,01), whilst 14 genes were over-expressed (FC≥+1,9, p<0,01) (Table S2). Note that of the 62 tRNA genes detected in our microarray analysis, only 6 were under-expressed (Fold change FC≤−1,9, p≤0,03). An in-depth statistical analysis established that the mis-regulated genes were all placed within a very specific genomic context (see Text S1 and Figure S7 in the supplementary data). Thus lack of Swd2.2 has only a minor impact on the expression of protein-coding transcripts (61 out of 5175 mRNA-coding genes). Importantly, no known regulator of chromosome condensation was found among the 61 mis-expressed genes.

### Lack of Swd2.2 has a context-dependent impact on transcription termination

Swd2, the budding yeast homologue of Swd2.2, is part of the APT sub-complex of the CPF [25]. In the absence of APT, RNA polymerase II transcribes longer transcripts, especially at snoRNA genes [26]. We investigated the role of Swd2.2 in the termination of transcription. Our strand-specific tiling arrays suggested that transcription failed to terminate properly at about 800 genes in the absence of Swd2.2 (see Methods and Table S3). Remarkably, the vast majority of genes with transcription termination defects were convergent, overlapping genes (Figure S8), suggesting that the requirement for Swd2.2 for transcription termination is context-dependent. Note however that we did not detect a stronger enrichment of Swd2.2 at those convergent genes requiring Swd2.2 for their transcription termination (Figure S9). Our microarray analysis failed to detect significant transcription termination defects at snoRNA genes. We confirmed this using a targeted RT-PCR approach [24] and showed that the transcription profile of specific snoRNAs genes was not significantly affected in the absence of Swd2.2 (Figure S10). Taken together, these data show that the reduced association of the CPF with chromatin that results from lack of Swd2.2 had only a limited and context-dependent impact on gene expression.

### Swd2.2 facilitates the association of Protein Phosphatase 1 PP1^Dis2^ with chromatin

In human cells, a homologue of Swd2.2, Wdr82, associates with Protein Phosphatase 1 (PP1) and its cofactor PNUTS as part of the PP1/PTW complex [27]. Blast searches highlighted significant homologies between human, xenopus PNUTS and the N-terminus of the uncharacterized fission yeast ORF SPCC74.02c (Figure S11). Interestingly, SPCC74.02c was previously shown to co-purify with Swd2.2 [23]. Based on this homology and results presented below, we propose that SPCC74.02c is the fission yeast homologue of PNUTS and we have therefore renamed it “Ppn1” for “**P**ombe **Pn**uts **1**”.

**Figure 4:**
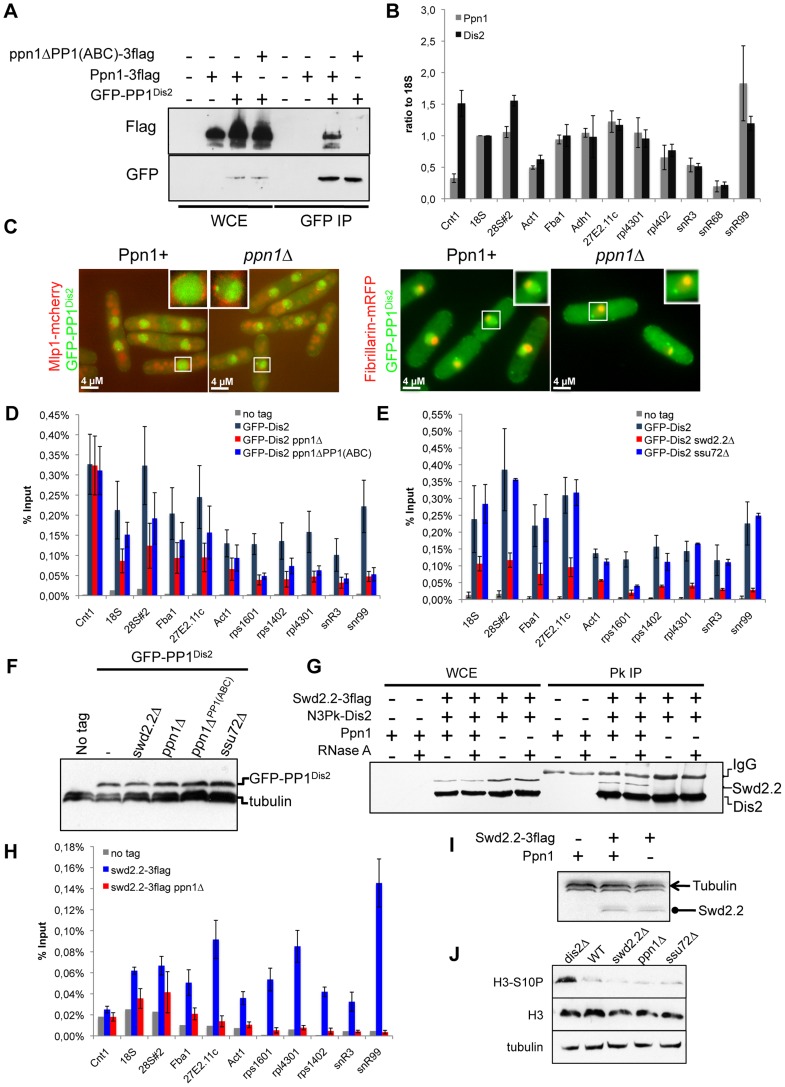
Swd2.2 facilitates the localization of PP1 phosphatase by interacting with the PNUTS homologue Ppn1. **A.** The interaction of Ppn1 with PP1^Dis2^ requires the three PP1-binding sites of Ppn1. GFP-tagged PP1^Dis2^ was immuno-precipitated from cycling cells in the presence of Flag-tagged Ppn1 (Ppn1-3flag) or Flag-tagged Ppn1 lacking the three PP1-binding sites (*ppn1ΔPP1(ABC)*-3flag). Whole cell extracts (WCE) and the immuno-precipitated material (GFP IP) were analyzed by western blot. **B.** Asynchronous populations of the indicated strains (Ppn1-3flag or GFP-PP1^Dis2^) were grown at 30°C and ChIP-qPCR was performed to analyze their enrichment at various sites along chromosomes. Enrichments were normalized to the values obtained at the RNA Polymerase I-transcribed 18S (mean ± standard deviation from 3 biological replicates). **C.** Lack of Ppn1 disrupts the nuclear localisation of GFP-tagged PP1^Dis2^. GFP-PP1^Dis2^ was imaged in dividing cells co-expressing (left panel) the mCherry-tagged nuclear envelope marker Mlp1 or (right panel) the mRFP-tagged nucleolar marker Fib1. **DE.** Asynchronous populations of the indicated strains were grown at 30°C and ChIP-qPCR was performed (mean ± standard deviation from 6 biological replicates). **F.** The protein stability of GFP-PP1^Dis2^ was assessed by western blot in the various mutant backgrounds used in **DE**. Tubulin was used as a loading control. **G.** The interaction between Flag-tagged Swd2.2 and Pk-tagged PP1^Dis2^ was analyzed by co-immunoprecipitation in protein extracts prepared from cycling cells in the presence or absence of Ppn1. Protein extracts were treated or not with RNase A prior to immuno-precipitation. Whole cell extracts (WCE) and the immuno-precipitated material (GFP IP) were analyzed by western blot. **H.** Asynchronous populations of the indicated strains were grown at 30°C and ChIP-qPCR was performed to analyze their enrichment at various sites along chromosomes. **I.** The protein stability of Flag-tagged Swd2.2 in the presence or absence of Ppn1 was assessed by western blot. Tubulin was used as a loading control. **J.** Asynchronous populations of the indicated strains were grown at 30°C. Protein extracts were prepared and analyzed by western blot using the indicated antibodies.

**Figure 5:**
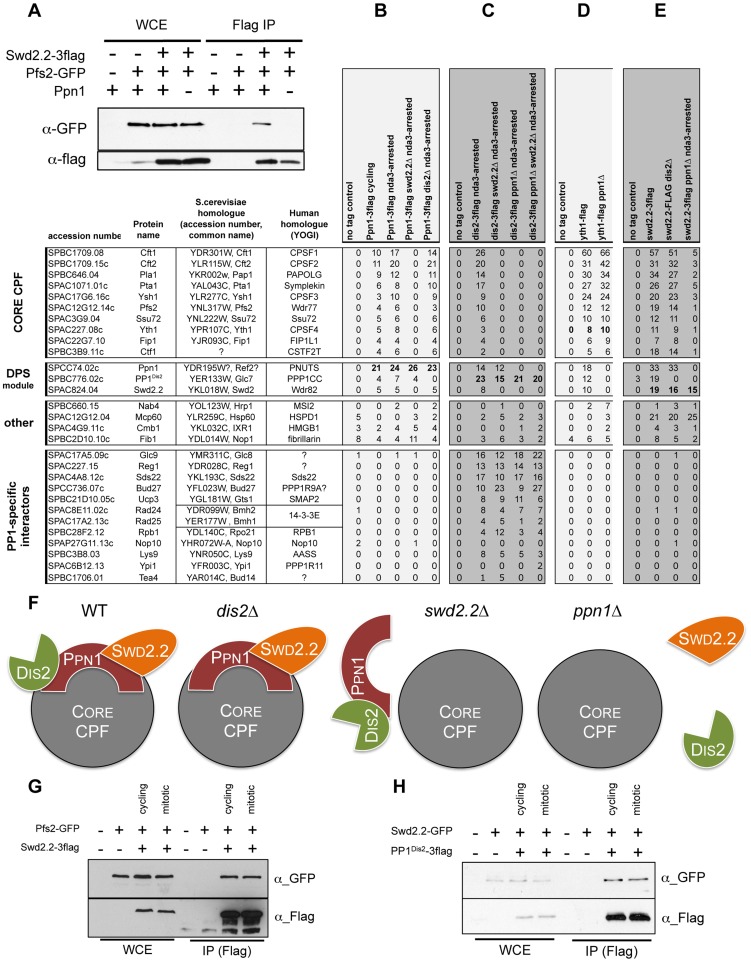
Swd2.2, Ppn1 and PP1^Dis2^ associate as a protein module to the CPF. **A.** The interaction between Flag-tagged Swd2.2 and GFP-tagged Pfs2 was analyzed by co-immunoprecipitation in protein extracts prepared from cycling cells in the presence or absence of Ppn1. Whole cell extracts (WCE) and the immuno-precipitated material (GFP IP) were analyzed by western blot. **BCDE.** The proteins indicated at the top were purified by affinity and their associated partners were identified by MS/MS mass-spectrometry analysis (see Methods). The number of unique peptides recovered for each protein is indicated. **F.** Scheme summarizing the proteomic data. **GH.** Cells expressing the indicated epitope-tagged proteins were synchronized in early mitosis (mitotic) or not (cycling), using the cold-sensitive *nda3KM311* mutation [33]. **G.** Flag-tagged Swd2.2 was immuno-precipitated to look at its interaction with the core CPF component Pfs2. **H.** Flag-tagged PP1^Dis2^ was immuno-precipitated to look at its interaction with the DPS component Swd2.2.

Ppn1 contains three amino acids motifs known to mediate a direct interaction with PP1 [28] (Figure S11). Co-immunoprecipitation experiments showed that Ppn1 did indeed interact *in vivo* with fission yeast PP1^Dis2^. This interaction required the three PP1-binding motifs of Ppn1 (Figure 4A). Interestingly, Ppn1 did not interact with the other fission yeast PP1 isoform, PP1^Sds21^, even in the absence of PP1^Dis2^ (*dis2Δ*) (see below, Figure 5B). ChIP experiments indicated that PP1^Dis2^ and Ppn1 have largely overlapping localization patterns, except at kinetochores (cnt1), where PP1^Dis2^ was comparatively 5 times more abundant than Ppn1 (Figure 4B). This is consistent with previous observations that PP1^Dis2^ has specific, kinetochore-based loading mechanisms [29]. Lack of Ppn1 severely disrupted the nuclear localization pattern of PP1^Dis2^, which became enriched in the Fib1-stained nucleolus [30] (Figure 4C). Consistent with this, ChIP analysis showed that the association of PP1^Dis2^ with chromatin was reduced although not completely abolished in the absence of Ppn1 or when the three PP1-binding motifs of Ppn1 were mutated (p<0,01 at all sites tested except at cnt1 where p = 0,93, Mann-Whitney test on 6 biological replicates, Figure 4D). These observations are consistent with the idea that the Ppn1-PP1^Dis2^ complex represents a major chromatin-associated PP1 activity in fission yeast.

As Ppn1 co-purified with Swd2.2 [23], we wondered whether Swd2.2 was also required for the association of PP1^Dis2^ with chromatin. ChIP experiments indicated that the association of PP1^Dis2^ with chromatin was indeed significantly reduced in the absence of Swd2.2 (p<0,01 Mann-Whitney test on 6 biological replicates) but not in the absence of the CPF-associated Ssu72 phosphatase (Figure 4E). Note that none of the mutations we tested affected the protein stability of PP1^Dis2^ (Figure 4F).

Co-immunoprecipitation experiments showed that Ppn1 but not RNA was required for the interaction between Swd2.2 and PP1^Dis2^ (Figure 4G). ChIP analysis indicated that Ppn1 was required for the association of Swd2.2 with chromatin but not for its stability (Figures 2A and 4H&I). Taken together, these observations suggested that Swd2.2, Ppn1 and PP1^Dis2^ form a complex in fission yeast related to the PTW/PP1 complex of vertebrates, and that this complex is important for the proper loading of PP1 phosphatase on chromosome arms.

In budding yeast, PP1^Glc7^ targets phospho-Ser10 on histone H3 for dephosphorylation [31] and the increased phosphorylation of Ser10 on histone H3 in some RNA processing mutants was recently shown to be associated with an increased compaction of the chromatin [32]. We wondered whether the partial rescue of the temperature sensitivity and chromosome segregation defects of *cut3-477* by *swd2.2Δ* could stem from the failure by PP1^Dis2^ to dephosphorylate Ser10 on histone H3. Western blot analysis indicated that lack of PP1^Dis2^ (*dis2Δ*) resulted indeed in the increased phosphorylation of Ser10 on histone H3 (Figure 4J). However, the levels of phosphorylated Ser10 on histone H3 were not altered in the absence of Swd2.2 or Ppn1 (Figure 4J). These observations were consistent with the idea that the pool of PP1^Dis2^ that we still detected on chromatin in the absence of Swd2.2 or Ppn1 was sufficient to antagonize the phosphorylation of Ser10 on histone H3. Alternatively, PP1^Dis2^ could dephosphorylate Ser10 on histone H3 even when it is not stably associated with chromatin. Whatever the explanation, our data show that the partial rescue of *cut3-477* by *swd2.2Δ* cannot be explained by the failure of PP1^Dis2^ to dephosphorylate Ser10 on histone H3.

### Ppn1 also interacts with the pre-mRNA 3′ end processing machinery

The data presented above established that Swd2.2, Ppn1 and PP1^Dis2^ co-localize and interact functionally. It remained unclear however whether they interacted within the CPF, or as part of an independent protein complex. Co-immunoprecipitation experiments showed that Ppn1 was required for the interaction between Swd2.2 and the Pfs2 (Figure 5A and see below). This was a first indication that their function was indeed connected to the CPF.

To establish whether Ppn1 and PP1^Dis2^ were genuine CPF components, we first purified Ppn1 by affinity and identified its associated proteins by mass-spectrometry analysis (see Methods). This approach showed that Ppn1 co-purified with PP1^Dis2^ but not PP1^Sds21^ (Figure 5B) and that it interacted with the 13 sub-units of the CPF. This interaction was found both in interphase and in early mitotic cells, synchronized at the metaphase to anaphase transition using the cold-sensitive tubulin mutation *nda3KM311* [33]. Similarly, PP1^Dis2^ but not PP1^Sds21^ co-purified with the 13 sub-units of the CPF (Figure 5C and Figure S12A). This showed that Ppn1, PP1^Dis2^ and Swd2.2 are genuine CPF components.

We had established that Swd2.2 was not required for the interaction between the CPF sub-units Pfs2 and Yth1 (Figure 3B). Here we sought to establish the importance of Ppn1 for CPF formation. Yth1 was affinity purified from wild-type and *ppn1Δ* mutants and its associated proteins identified by mass-spectrometry analysis (Figure 5D). A typical example of such purifications is shown on Figure S12B. In wild-type cells, Yth1 co-purified with all known CPF sub-units, including Swd2.2, Ppn1 and PP1^Dis2^. In the absence of Ppn1, Swd2.2 and PP1^Dis2^ dissociated from the CPF whilst the other CPF sub-units remained bound to Yth1 (Figure 5D). Similarly, lack of Swd2.2 triggered the dissociation of Ppn1 and PP1^Dis2^ from the CPF but of no other CPF sub-units (Figure S12A). These observations suggested that Ppn1, Swd2.2 and PP1^Dis2^ constitute an independent module within the CPF, whose loss does not alter CPF integrity.

To confirm this, we purified PP1^Dis2^, Swd2.2 and Ppn1 and identified their binding partners by mass-spectrometry analysis. PP1^Dis2^ bound to all CPF sub-units and various other proteins (Figure 5C). In the absence of Swd2.2, PP1^Dis2^ no longer bound to the CPF but remained associated with Ppn1 and its other binding partners. In contrast, when Ppn1 was missing, PP1^Dis2^ failed to bind to Swd2.2 and the CPF but remained associated with its other binding partners. The interaction between Swd2.2 and the CPF required Ppn1 (Figure 5A&E) and conversely, the interaction between Ppn1 and the CPF required Swd2.2 (Figure 5B). Thus Swd2.2 and Ppn1 are inter-dependent for their association with the CPF and both are necessary for the association of PP1^Dis2^ with the CPF. These observations are summarized on Figure 5F. Collectively, these data establish that PP1^Dis2^, Ppn1 and Swd2.2 form a protein module associated with the CPF whose absence does not affect significantly the composition of the core CPF. We named this module the DPS (**D**is2-**P**pn1-**S**wd2.2) module. Note that to further illustrate the quality of our affinity purifications, we provide the full list of unique peptides recovered after purifications of Yth1, Swd2.2, Ppn1 and PP1^Dis2^ ranked by abundance (Table S4).

Figure 5B shows that the interaction between Ppn1 and the CPF was detectable whether protein extracts were prepared from cycling or early mitotic cells. This suggested that DPS interacts with the CPF throughout the cell-cycle. We sought to confirm these observations using co-immunoprecipitation approaches. Here we show that synchronizing cells in early mitosis did not alter complex formation between Swd2.2 and the CPF sub-unit Pfs2 (Figure 5G), nor did it alter the interaction between Swd2.2 and PP1^Dis2^ (Figure 5H). Taken together, these data show that DPS is stable and remains associated with the CPF in early mitosis. Similarly, ChIP analysis indicated that the levels of chromatin-associated CPF remained comparable in cycling cells and in early mitotic cells (Figure S13). Taken together, these data show that DPS is present on chromatin throughout the cell-cycle. This is consistent with the idea that DPS can antagonize the action of condensin in mitosis.

### DPS opposes condensin-mediated chromosome condensation

**Figure 6:**
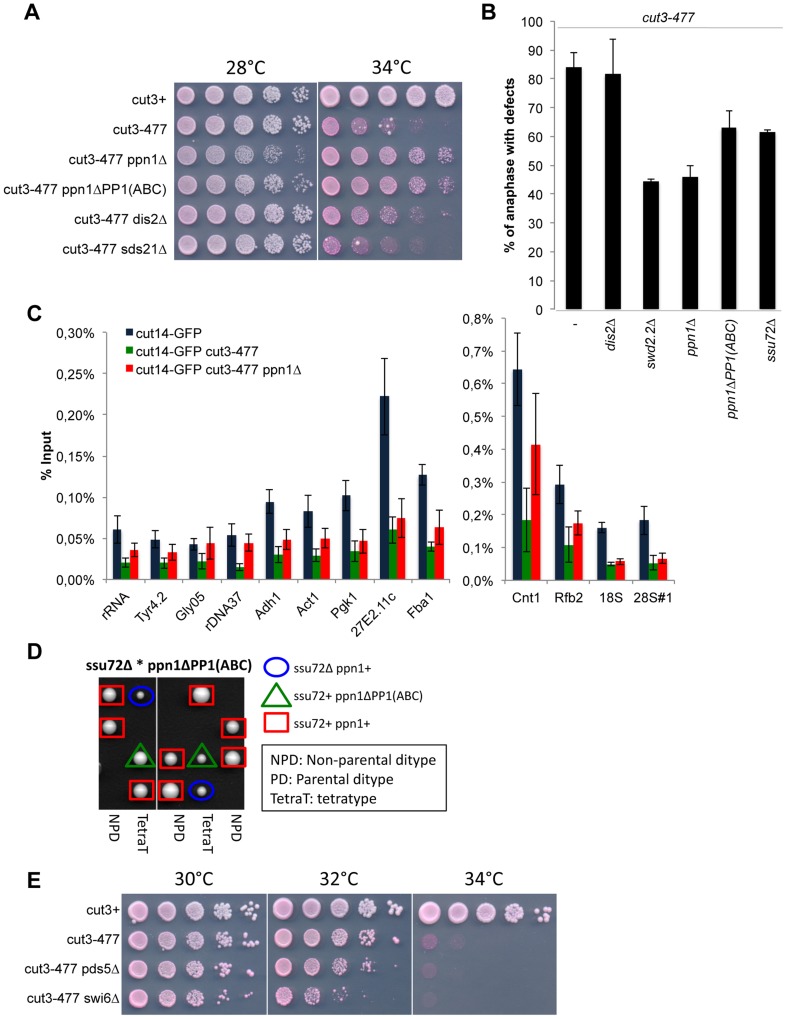
Ppn1 and Ssu72 oppose condensin-mediated chromosome condensation. **A.** Serial dilutions of the indicated strains were plated on rich media at the indicated temperatures. **B.** Chromosome segregation in anaphase was visualized after growing cells of the indicated genotypes for one generation at 34°C. Anaphases were scored as defective when lagging chromatin was detected between the two main DNA masses. For each genotype, a minimum of 6 independent experiments was performed in which a minimum of 100 anaphase cells was scored. **C.** The indicated strains were grown at 34°C for 3 hours and ChIP-qPCR was performed to analyze the amount of Cut14-GFP cross-linked to chromatin (mean ± standard deviation from 8 biological replicates). See text for details for the statistical analysis of the experiments. **D.** Tetrad dissection was used to show that the double mutant *ssu72Δ ppn1ΔPP1(ABC)* is dead. **E**. Serial dilutions of the indicated strains were plated on rich media at the indicated temperatures.

If DPS is indeed a functional module, the mutation of all its components should give similar phenotypes. To validate this prediction, we tested whether Ppn1 and PP1^Dis2^, like Swd2.2, could oppose condensin-mediated chromosome condensation. This is indeed what we observed. Lack of Ppn1 (*ppn1Δ*) significantly improved the growth of *cut3-477* cells at the restrictive temperature (p<0,01, Mann-Whitney test; Figure 6A). The poor growth of *cut3-477* at the restrictive temperature was also improved when Ppn1 lost its ability to bind PP1^Dis2^, suggesting that the negative effect of Ppn1 on *cut3-477* is mediated, at least in part, by its interaction with PP1^Dis2^ (p<0,01, Mann-Whitney test). Note however that lack of the core CPF component Ctf1 had no effect on the growth of *cut3-477* cells (Figure S14A). Similarly, lack of Ppn1 but not of Ctf1 significantly decreased the percentage of defective anaphases (Figure 6B and Figure S14B), showing that lack of Ppn1 could restore the formation of segregation-competent chromosomes in a majority of mitotic *cut3-477* cells. Finally, ChIP experiments showed that lack of Ppn1, like lack of Swd2.2, also improved the localization of condensin in *cut3-477* cells (p value <0,01, Mann-Whitney test on 8 biological replicates, Figure 6C).

### DPS is redundant with Ssu72

Lack of PP1^Dis2^ (*dis2Δ*) had a weaker suppressive effect on *cut3-477* than either *swd2.2Δ* or *ppn1Δ* (Figure 6AB). To explain this observation, we speculated that another phosphatase could at least partly substitute for PP1^Dis2^. Here we present evidence that one such phosphatase could be the CPF-associated Ssu72 phosphatase. First, lack of Ssu72 exhibited a mild suppressive effect on *cut3-477* (p = 0,05 Mann-Whitney test, Figure 6B), although it did not improve significantly the localization of condensin in *cut3-477* cells (Figure S15). Secondly, we found that the double mutant *ssu72Δ ppn1ΔPP1(ABC)*, which lacks CPF-associated phosphatases, is dead (Figure 6D). This evidence suggests that both CPF-associated phosphatases PP1^Dis2^ and Ssu72 regulate condensin-dependent chromosome condensation. Furthermore, they act redundantly within the CPF to perform one or more essential function(s).

We wondered what could be the substrates of CPF-associated phosphatases whose hyper-phosphorylation in the absence of DPS could facilitate the function of condensin. We have previously ruled out that histone H3 could be a relevant target (Figure 4J). Human Ssu72 was shown recently to interact with the condensin-related cohesin complex and thereby facilitate sister-chromatid cohesion [34]. This opened the possibility that a weakened cohesin function in *ssu72Δ* or *dps* mutants could facilitate the function of condensin in *cut3-477* cells. To test this, we weakened chromosome cohesion by mutating two known regulators of cohesin, Swi6 (*swi6Δ*) and Pds5 (*pds5Δ*). Neither deletion was able to suppress the growth defect of *cut3-477* (Figure 6E). This suggested that a weakened cohesion in *ssu72* or *dps* mutants is not sufficient to explain their ability to suppress *cut3-477*.

## Discussion

Here we provide evidence that the CPF sub-unit Swd2.2 is a negative regulator of condensin-mediated chromosome condensation. We show that Swd2.2 is part of a PP1-containing protein module associated with the CPF, whose absence does not alter CPF organization. We named this module the DPS module. Lack of DPS destabilized mildly the association of the CPF with chromatin but the resulting defects in gene expression remained relatively minor and context-specific. However, we show that DPS and the CPF-associated Ssu72 phosphatase oppose condensin-mediated chromosome condensation. Their relevant substrate(s) in this process is(are) still unknown, although we have shown that it is unlikely that it could be phospho-Ser10 on histone H3, cohesin or Aurora B-dependent phosphorylation of Cnd2. However at this stage, our data do not exclude the possibility that DPS and Ssu72 target other phosphorylated sites on condensin.

### DPS, a conserved regulator of chromosome condensation

Human PNUTS is a scaffold for the PP1-containing PTW/PP1 complex, where it interacts directly with three proteins, Wdr82, Tox4 and PP1 [27]. Our data strongly suggest that Swd2.2 is the fission yeast functional homologue of Wdr82 and Ppn1 the homologue of PNUTS. We could not identify a homologue of Tox4 in fission yeast. Human PNUTS, like Ppn1, co-purified with the pre-mRNA 3′ processing complex [35]. As such, PTW/PP1 is reminiscent of the DPS complex we identified here.

Interestingly, we note that the other PP1 isoform in fission yeast, PP1^Sds21^ was not able to replace PP1^Dis2^ within the DPS. Furthermore, the nuclear localization pattern that PP1^Dis2^ adopted in the absence of Ppn1 was highly similar to the nuclear localization pattern that was reported for PP1^Sds21^ [36]. This shows that the ability to bind Ppn1 and the CPF is a major distinguishing feature between the two isoforms of PP1 in fission yeast. The structural features that allow PP1^Dis2^ but not PP1^Sds21^ to bind to Ppn1 remain to be elucidated.

In the human PTW/PP1 complex, the interaction between hPNUTS, Wdr82 and PP1 is likely to be relevant for chromosome condensation, as the domain of hPNUTS that mediates the interaction with both PP1 and Wdr82 is sufficient to induce chromosome decondensation *in vitro* [27], [37]. This suggests that the function of DPS as a negative regulator of chromosome condensation is likely to be evolutionary-conserved.

### A role for DPS at Pol III-transcribed genes?

Our data show that lack of Swd2.2 had a significant impact on the association of the RNA Pol III transcription machinery with chromatin. In our hands, the effects of *swd2.2Δ* on the recruitment of the Pol III machinery were highly reminiscent of the effects of mutating the TFIIIC component Sfc3 [6]. Strikingly, *sfc3-1* is, like *swd2.2Δ*, a suppressor of *cut3-477* [6]. Unfortunately, we repeatedly failed to generate viable strains with a reduced expression of TFIIIC. As a consequence, we could not establish whether the reduced association of TFIIIC with chromatin observed in *swd2.2Δ* and *sfc3-1* cells could by itself facilitate the localization of condensin. Our observations are however consistent with the idea put forward previously that the local reduction in RNA polymerase activity can facilitate the association of condensin [10].

Part of the effects of *swd2.2Δ* on the recruitment of the Pol III machinery is likely to be indirect, because lack of Swd2.2 had a mild impact on the expression of Sfc6, a component of TFIIIC. On the other hand, we detected a small but significant enrichment of Swd2.2 and Pfs2 at Pol III-transcribed genes, consistent with the idea that it could also play a direct role there. The fact that a factor associated with the RNA Pol II RNA processing machinery such as Swd2.2 could also play a role at Pol III genes is not unprecedented. In budding yeast in particular, the Nrd1/Nab3 complex is involved in processing both Pol II and Pol III transcripts [38] and Pcf11 in *S.cerevisiae* is enriched at Pol III genes [39]. Interestingly, budding yeast PP1 was shown to dephosphorylate Sen1 helicase, which associates with Nrd1/Nab3 [26] and we identified Sen1 as a suppressor of *cut3-477* (Table S1). Whether or not Swd2.2 is involved in directing the activity of PP1 towards Sen1 at Pol III genes in fission yeast will require further studies.

### (CTD) Phosphatases and condensation

In chicken DT40 cells, preventing the association of PP1 with chromatin was sufficient to rescue anaphase chromosome segregation when condensin was deficient [40]. Our data are consistent with these observations. Earnshaw and colleagues proposed that preventing the association of PP1 with chromatin resulted in the hyper-activation of “Regulator of Chromosome Architecture” (RCA), a hypothetical condensin-independent chromosome compacting activity [40]. Our stories differ on that point, as we show that lack of Swd2.2 and Ppn1 impacted the localization of condensin in *cut3-477* cells, indicating that the target of DPS is ultimately condensin and not RCA.

Cdc14 phosphatase in budding yeast was shown to dephosphorylate the CTD domain of RNA Pol II [10]. When Cdc14 activity was impaired, RNA Pol II-dependent transcription occurred abnormally at repetitive sequences and condensin was displaced from these sequences [10]. It was concluded from these data that CTD dephosphorylation participates in the inactivation of RNA polymerase II in a way that facilitates the binding of condensin and the assembly of a condensed chromosome. Here, our work shows that PP1 and Ssu72, which, like Cdc14, have been shown to target the CTD domain for dephosphorylation [41], [42], [43], are negative regulators of condensin. Further studies are required to understand this apparent discrepancy.

We note however that, in budding yeast, lack of Ssu72 interferes with transcription elongation [44]. Interestingly, the N-terminus domain of Ppn1 shows homology to TFIIS (Figure S11), whose absence impairs transcription elongation in fission yeast [45]. This opens the possibility that Ppn1 could also modulate transcription elongation. We recently identified as a suppressor of *cut3-477* a mutation in the Mediator sub-unit Nut2 [46] and our present screen also identified the deletion of the TFIIS homologue Tfs1 (*tfs1Δ*) as a suppressor of *cut3-477* (Table S1). Taken together, these observations reinforce the idea of complex functional links between condensin and transcription and suggest that reducing the processivity of RNA Polymerase II or III could somehow facilitate the function of condensin. The mechanisms involved remain to be elucidated.

## Materials and Methods

### Fission yeast strains

A list of the strains used in this study is given in Table S5. Standard genetic crosses were employed to construct all strains. Yeast cells were grown according to standard procedures. Dis2-3flag, Yth1-3flag, Swd2.2-3flag, Ppn1-3flag, Yth1-GFP, Swd2.2-GFP, *ppn1Δ*, *swd2.2Δ*, *rtt103Δ* were generated using a standard PCR procedure. To obtain *ppn1ΔPP1(ABC)*-3flag, the C-terminus of Ppn1 was PCR amplified and cloned into pGEMT-easy (Promega, Madison, Wisconsin, USA). Site-directed mutagenesis was then used to mutate the three PP1-binding sites using Quickchange protocols (Stratagene). Overlapping PCR was used to add a 3xFlag tag and a cassette of resistance to nourseothricin (NatR) to the C-terminus of Ppn1. This PCR product was used to transform yeast. Proper integrants were selected by PCR and western blot and were sequenced to verify the presence of the mutations.

### Immunoprecipitation

2.10^8^ cells were frozen in liquid nitrogen and broken open in lysis buffer (50 mM HEPES [pH 7.6], 75 mM KCl, 1 mM MgCl2, 1 mM EGTA, 0.1% Triton, 1 mM sodium vanadate, microcystin, 1 µg/mL leupeptin, 1 µg/mL pepstatin, 1 µg/mL chymostatin, and 1 mM Pefabloc) using zirconium beads and a Fast-prep machine (40″, 6,5 ms^−1^, 3 times). Clarified extracts were then incubated for 60 min at 4°C with Protein A-coupled Dynabeads incubated previously with the proper antibody according to the manusfacturer's recommendations. The immunoprecipitated complexes were washed three times with lysis buffer and once with phosphate-buffered saline containing 0.02% Tween 20. Immunoprecipitated complexes were analyzed by immunoblot using a semi-dry transfer protocol. Cnd2 phosphorylation was analysed as described previously [2].

### Protein purification

5 g of cells were broken using a Retsch MM400 mill for 180 s at 30 Hz (repeat three times). The broken cells were then resuspended in lysis buffer (50 mM Hepes-KOH [pH 7.6], 100 mM KCl, 1 mM EGTA, 10% Glycerol, 0.1% NP40, 1 mM MgCl_2_, 1 µg/mL leupeptin, 1 µg/mL pepstatin, 1 µg/mL chymostatin, and 1 mM Pefabloc) and then sonicated twice for 30 seconds on ice (1.2 W per mL). After centrifugation (5′, 4°C, 4000 rpm), the supernatant was filtered through a 2.7 µM then a 1.6 µM glass microfiber filter (Whatmann 25 mm GD/X). 25 µL of 50/50 slurry of anti-Flag M2 affinity agarose beads (Sigma) was added and incubated on a rotating wheel at 4°C for 30′. The beads were then washed three times in cold lysis buffer and then twice in 50 mM Hepes-KOH [pH 7.6], 50 mM KCl. Proteins were eluted by two 5′ incubations in 50 µL of 50 mM H3PO4.

### Mass-spectrometry analysis

All chemicals were purchased from Sigma-Aldrich (UK) unless otherwise stated. Acetonitrile and water for LC-MS/MS were HPLC grade (Fisher, UK). Formic acid was Suprapure 98-100% (Merck, Darmstadt, Germany) and trifluoroacetic acid was 99% purity sequencing grade. All HPLC-MS connector fittings were from Upchurch Scientific or Valco (Hichrom and RESTEK, UK).

Gel-free samples were prepared and loaded onto strong cation exchange columns, reduced with DTT, alkylated with iodoacetamide, and digested with trypsin as described in [47]. Samples were dried under low pressure and reconstituted in 10 µl of 0.1% (v/v) formic acid 2.5% (v/v) acetonitrile for nano-LC-MSMS. Capillary-HPLC-MSMS data were acquired on an on-line system consisting of a micro-pump (1200 binary HPLC system, Agilent, UK) coupled to a hybrid LTQ-Orbitrap XL instrument (Thermo-Fisher, UK). The LTQ was controlled through Xcalibur 2.0.7. HPLC-MS methods have been described previously [48].

### Protein identification and quantification

MS/MS data were searched using MASCOT Versions 2.3 (Matrix Science Ltd, UK) against a Schizosaccharomyces pombe database downloaded from the Sanger Institute (http://www.sanger.ac.uk/) with 5022 sequences. Variable methionine oxidation, STY phosphorylation, protein N-terminal acetylation and fixed cysteine carbamidomethylation were used in all searches. Precursor mass tolerance was set to 7 ppm and MS/MS tolerance to 0.4 amu. The significance threshold (p) was set below 0.05 (MudPIT scoring in Mascot). All LC-MS runs were combined using MaxQuant (version 1.0.13.8), assuming a false positive rate of 0.01 [49].

### RNA extraction and transcription termination assay

RNA was extracted from 2.10^8^ cells according to the procedure described in [50]. qPCR were performed a Rotorgene machine (Qiagen). Retrotranscription and qPCR were performed as described previously [51]. Briefly, cells were grown at 34°C for two hours, and total RNA extracted. Total RNA was reverse-transcribed using random hexamers and cDNAs amplified by PCR using the indicated primers. PCR products were stained with Sybr Green and quantified in gel using the FLA-5000 imaging system (Fujifilm).

### Strand-specific tiling arrays

ProfilXpert (http://www.profilexpert.fr/) performed the strand-specific tiling arrays and their initial analysis. The data were established in triplicates. RNAs extracted in the lab were purified using the miRNeasy mini kit (Qiagen), dosed using a nanodrop and their quality was assessed using a Bioanalyzer 2100 (Agilent). To prepare cDNAs, 250 ng of RNA were then amplified using the WT Expression Kit (Ambion). The resulting cDNAs were then dosed (Nanodrop) and their quality assessed using a Bioanalyzer 2100 (Agilent). 5.5 µg of cDNAs were then fragmented and labelled using the GeneChIP WT Terminal Labelling Kit (Affymetrix). 5 µg of cDNAs were then hybridized on a GeneChip S.pombe Tiling 1.0 Array (Affymetrix). The data were normalized using Quantile Normalization [52] and RMA background correction [53] using the software Partek Genomics Suite 6.5. To establish which genes were deregulated in *swd2.2Δ*, the median value of the signals recorded for each gene in each replicate was first calculated. These median values were then averaged for the three replicates. For each gene, The Fold-change (FC) and the corresponding p-value were calculated to express variation in expression between *swd2.2Δ* and swd2.2+ cells. To identify genes with potential transcription termination defects, we proceeded as follows: for every gene whose 3′UTR is annotated in the fission yeast database, we calculated the ratio between the average signal recorded in the 3′UTR and the average signal recorded in the ORF in *swd2.2+* and *swd2.2Δ* cells. We considered that genes had a transcription termination defect in cells lacking Swd2.2 when the ratio obtained for *swd2.2Δ* was at least 1.5 greater than the ratio obtained in *swd2.2+*. The data can be accessed on GEO under the accession number GSE38005.

### Statistical analysis of genomic features

All statistical analysis was done using the R software (www.r-project.org). Genomic features annotations were downloaded on the 09/05/2011 from the Sanger centre (ftp://ftp.sanger.ac.uk/pub/yeast/pombe/GFF/). To test for differential expression between *gDWN-1* and *gDWN* genes in the wild-type context, we computed the 47 logratios and tested their departure from zero using a Wilcoxon test. To test if the intergenic distance (IGR) between *gDWN-1* and *gDWN* was particularly short or long compared to any other gene, we compared the median value of the 47 observed IGR to the distribution of median values obtained from 10,000 randomly chosen sets of 47 genes in the genome. The resulting empirical null distribution is shown on Figure S7C. The median IGR observed from the actual set of 47 *gDWN* genes was shorter than all simulated median IGRs. The statistical significance for claiming that these IGRs are particularly short is therefore lower than 1/10,000. Similarly, IGRterm distances observed on the set of 780 *gTERM* genes were computed and their median was compared to the distribution of median values from 10,000 sets of 780 genes picked at random from the genome (empirical null distribution of Figure S8C). Since no median value of the random sets was as small as the observed one, the claim for short IGRterm values of *gTERM* genes is supported at a significance lower than 1/10,000.

### Chromatin immunoprecipitation

10^8^ cells were treated with 1% formaldehyde (Sigma) at 18°C for 30 mn. After extensive washes with cold PBS, cells were frozen in liquid Nitrogen. Frozen cells were then broken open in cold lysis buffer (Hepes-KOH 50 mM [pH 7.5], NaCl 140 mM, EDTA 1 mM, Triton 1%, Na-deoxycholate 0.1%, PMSF 1 mM) using Acid-wash Glass beads (Sigma) and a Fast-Prep machine (6 times 1′ at 6,5 ms^−1^). The lysats were then sonicated at 4°C using a Diagenode sonicator. Immuno-precipitation was done overnight at (4°C) using ProtA-coupled Dynabeads previously incubated with the anti-GFP A11122 antibody (Invitrogen). Beads were washed successively with (10′ incubation on rotating wheel): Wash I buffer (20 mM Tris pH 8, 150 mM NaCl, 2 mM EDTA, 1% Triton-X100, 0.1% SDS), Wash II buffer (20 mM Tris pH 8, 500 mM NaCl, 2 mM EDTA, 1% Triton-X100, 0.1% SDS) and Wash III buffer (20 mM Tris pH 8, 1 mM EDTA, 0.5% Na-deoxycholate, 1% Igepal, 250 mM LiCl). After two additional washes in TE pH 8, the cross-links were reversed by incubation at 65°C (6 hours minimum) with elution buffer (TE pH 8, SDS 0.5%, 0.35 mg/mL Proteinase K). The immuno-precipitated DNA was then purified using the Wizard PCR purification kit (Promega) according to the manusfacturer's instructions. The DNA was then analyzed by qPCR. Primers are available on demand.

## Supporting Information

Figure S1 Lack of Swd2.2 does not improve the viability of other mutants defective in chromosome architecture. **AB.** Serial dilutions of the indicated strains were plated on rich media at the indicated temperatures.
(TIF)

Figure S2 Lack of Swd2.2 does not alter the stability of Cut14 protein. Western blot analysis of the indicated strains.
(TIF)

Figure S3 Lack of Swd2.2 suppresses the synthetic lethal interaction between *cut3-477* and *mde4Δ*. Serial dilutions of the indicated strains were plated on rich media at the indicated temperatures.
(TIF)

Figure S4 Lack of Swd2.2 does not significantly alter the localization of the wild-type condensin complex. ChIP qPCR of the GFP-tagged condensin sub-unit Cut3 (Cut3-GFP) in the presence or absence of Swd2.2. Strains were grown in cycling conditions in rich medium at 30°C (mean ± standard deviation from 3 biological replicates).
(TIF)

Figure S5 Lack of Swd2.2 has no detectable effect on Aurora B-dependent Cnd2- phosphorylation or H2A.z^Pht1^ acetylation. **A.** Cycling or early mitotic cells (arrested in prometaphase with the cold-sensitive *nda3-KM311* mutation) expressing the condensin sub-unit Cnd2 tagged with GFP at its endogenous locus were imaged under the microscope to count the number of mitotic, GFP-positive cells. Protein extracts were prepared from the same cells and GFP-tagged Cnd2 was immuno-precipitated. The immuno-precipitated complexes were analyzed by western blot using an antibody recognising the phospho-modified form of Cnd2, as described previously [2]. The mitotic indexes in each population is indicated underneath, based on the number of GFP-positive cells in the population. **B**. The same experiment as in (**A**) was repeated with *cut3-477* or *cut3-477 swd2.2Δ* cells expressing GFP-tagged Cnd2 grown at the restrictive temperature of 34°C for one generation (3 hours). As the mitotic index is much smaller in these cells compared to *nda3KM311*-arrested cells, a diluted “*nda3*” samples was put on the same gel as control. **C.** Lack of Swd2.2 has no detectable effect on the acetylation of H2A.z^Pht1^. Protein extracts were prepared from cells expressing HA-tagged Pht1 and analyzed by western blot using an acetyl-specific antibody recognising the acetylated isoform of Pht1 (see [22]). A HA-tagged, non-acetylable version of Pht1 (Pht1-4KR) was used as a control.
(TIF)

Figure S6 The association of Sfc6 and Rpc25 at Pol III-transcribed genes is impaired in *sfc3-1* cells. **A**. Genotyping of the *sfc3-1* mutation. (left panel) Method: the *sfc3-1* mutation creates a BspH I site. For genotyping, a PCR product (fragment 806–1458 bp) is digested with BspH I for 2 hours. PCR products derived from sfc3+ cells remain undigested (right panel). Genotyping of the strains used for the ChIP in **B** and **C**. **BC**. ChIP qPCR of the indicated strains grown in cycling conditions for 2 hours at 36°C, the restrictive temperature of *sfc3-1* (mean ± standard deviation from 6 biological replicates).
(TIF)

Figure S7 Genomic context of the 47 down-regulated genes in cells lacking Swd2.2. **A.** Scheme explaining the genomic context of the 47 down-regulated genes. *gDWN*  =  gene of interest; *gDWN-1*  =  gene positioned on the same DNA strand directly upstream of g. IGR: Intergenic distance in bp between the end of the 3′UTR of *gDWN-1* and the beginning of the 5′UTR of *gDWN*. F_as_ Fraction of *gDWN* covered by an antisense transcript. **B.** Distribution of expression differences between *gDWN* and *gDWN-1* in the wild-type context. X-axis: log2 ratio of the expression level of *gDWN-1* over the expression level of *gDWN*. Y-axis: gene counts. Sums of all bars  = 47, representing all pairs (*gDWN-1*; *gDWN*) for the 47 *gDWN* genes under-expressed when Swd2.2 is absent. Red vertical line: median value. Green dotted line indicates the expected median if *gDWN-1* and *gDWN* had similar expression levels. **C.** The median IGR observed for the 47 *gDWN* genes (red line) is shorter than for other genes of the genome. 10,000 sets of 47 genes were drawn at random from the genome, and the median IGR computed for each set. The black histogram shows the distribution of the resulting 10,000 median values. All of them exceeded the value observed on the actual set of 47 *gDWN* genes. **D.** *gDWN* genes are more often covered by an antisense transcript than other genes of the genome. X-axis: fraction of gene covered by antisense transcription (F_as_). The yellow histogram shows the distribution of F_as_ values for the 47 *gDWN* genes. For comparison, the blue histogram shows the distribution of Fas values calculated for 10,000 random sets of 47 genes picked in the genome. The two distributions were significantly different (Kolmogorov-Smirnov *P*-value  = 0.0004). This diagram indicates that the majority of genes in the *S.pombe* genome are not covered by an antisense transcript (F_as_ = 0 for ∼60% of genes, blue histogram), whilst roughly 10% of genes are completely covered by an antisense transcript (F_as_ = 1, blue histogram). For the 47 *gDWN* genes, the percentage of genes entirely covered by an antisense transcript increases to roughly ∼35% (F_as_ = 1, yellow histogram). **E.** Tiling-array hybridization intensity profiles along two *gDWN* genes. **F.** The expression levels of candidate genes were verified by RT-qPCR in the indicated CPF mutants. The values presented are normalized to the expression levels of act1 established concomitantly. Results are the average of 3 biological replicates. Error bars represent standard deviation.
(TIF)

Figure S8 The effect of Swd2.2 on transcription termination is context-dependent. **A.** Tiling-array hybridization intensity profiles along two genes showing transcription termination defects when Swd2.2 is missing. Black boxes correspond to the coding region of the genes and grey boxes correspond to the UTRs. White boxes correspond to introns. The arrow indicates the orientation of transcription. **B.** Scheme explaining the genomic context of the genes with transcription termination defects in the absence of Swd2.2. *gTERM*  =  gene of interest showing no difference of microarray signal in the coding region but a stronger signal in the 3′UTR when Swd2.2 is missing. *gTERM+1RV*: the first downstream gene in the reverse orientation. IGR^term^: IGR regions located between *gTERM* and *gTERM*+1RV. **C.** The size of IGR^term^ was measured for each gene of interest. Red vertical line  =  median size measured for the 780 *gTERM* genes. It is negative, showing that most *gTERM* genes overlap with their immediate downstream reverse gene. Black histogram: distribution of median values obtained on 10,000 random sets of 780 genes.
(TIF)

Figure S9 Enrichment of Swd2.2 established by ChIP at convergent genes exhibiting transcription termination defects when Swd2.2 is missing. Rhp18 and Mde4 are two genes placed in situation of convergence with respectively SPBC1734.07c and Atg7, whose transcription termination is affected in the absence of Swd2.2. Fba1 and Pgk1 suffer no transcription termination defect in the absence of Swd2.2. ChIP analysis showed that Swd2.2 is not more abundant in the 3′UTR of Rhp18 and Mde4 compared to the 3′UTR of Fba1 and Pgk1 (mean ± standard deviation from 5 biological replicates).
(TIF)

Figure S10 Lack of DPS has a moderate effect on transcription termination at snoRNAs. Transcription termination was monitored in deletion mutants of various CPF-associated proteins at Ura4+ (**A**), Cdc18 (**B**), snoR99 (**C**) and snoR68 (**D**). Each panel follows the same organization: on the left, the tiling-array hybridization intensity profiles along the gene of interest is displayed for each strand as seen for Swd2.2+ or *swd2.2Δ* cells; on the right, the result of a qRT-PCR strategy designed to quantify the proportion of RNA that have been transcribed passed the previously-identified site of transcription termination. Termination sites are identified on the figure together with genomic features such as restriction sites. For each gene examined, the PCR product F-R1 served as internal loading control. The histograms show the size-normalized ratio to R1 =  (band intensity/band size)/(R1 band intensity/R1 size). For each, n = 3 biological replicates.
(TIF)

Figure S11 Ppn1 shows significant homology with human and xenopus PNUTS. **A.** Scheme explaining the domain organization of the PNUTS homologues in Human and *S.pombe*. The domain showing homology to TFIIS, and the PP1-binding consensus sites are highlighted. **B.** The PRALINE software (http://www.ibi.vu.nl/programs/pralinewww/) was used to create an alignment between the human and Xenopus PNUTS homologues and the fission yeast SPCC74.02c (Ppn1). The region of human PNUTS found to be sufficient to induce chromosome decondensation *in vitro* [37] is underlined in red. The PP1-binding motifs are underlined in black.
(TIF)

Figure S12 Mass-spectrometry analysis of Sds21- and Yth1-associated proteins. **A.** The proteins indicated at the top were affinity purified and the associated proteins identified by MS/MS mass-spectrometry analysis (see Methods). The number of unique peptides recovered for each protein is indicated. **B.** The proteins recovered after affinity purification of Flag-tagged Yth1 were run on an SDS-PAGE gel and visualized by coomassie staining.
(TIF)

Figure S13 The CPF sub-unit Pfs2 remains associated with chromatin in early mitotic cells. ChIP-qPCR analysis was performed to monitor the association of GFP-tagged Pfs2 with chromatin in cycling cells and in cells synchronized at the metaphase to anaphase transition using the cold-sensitive tubulin mutation *nda3KM311* (mean ± standard deviation from 6 biological replicates).
(TIF)

Figure S14 The CPF component Ctf1 does not oppose condensin-mediated chromosome condensation. **A**. Serial dilutions of the indicated strains were plated on rich media at the indicated temperatures. **B.** Chromosome segregation in anaphase was visualized after growing cells of the indicated genotypes for one generation at 34°C. Anaphases were scored as defective when lagging chromatin was detected between the two main DNA masses. For each genotype, 3 independent experiments were performed in which a minimum of 100 anaphase cells was scored.
(TIF)

Figure S15 Lack of Ssu72 does not restore the localization of condensin in *cut3-477* cells. The indicated strains were grown at 34°C for 3 hours and ChIP-qPCR was performed to analyze the amount of Cut14-GFP cross-linked to chromatin (mean ± standard deviation from 6 biological replicates). The enrichments of Cut14 observed in *cut3-477* and *cut3-477 ssu72Δ* cells were not significantly different (p>0,2, Wilcoxon - Mann Whitney).
(TIF)

Table S1 List of the gene deletions identified in our screen for suppressors of the growth defect of *cut3-477* at 34°C.
(XLSX)

Table S2 List of the genes whose expression is significantly altered in the absence of Swd2.2.
(XLSX)

Table S3 List of the genes exhibiting a transcription termination defect in the absence of Swd2.2.
(XLSX)

Table S4 List of the proteins identified after LC-MS/MS analysis of the affinity purifications of Yth1, Ppn1, Swd2.2 and PP1^Dis2^. Numbers correspond to the number of unique peptides identified for each protein. Proteins labeled in black were discarded as not specific as they were also identified after purification of a no-tag control.
(XLSX)

Table S5 List of the yeast strains used in this study.
(XLSX)

Text S1 Analysis of the genomic context surrounding the genes that are mis-expressed in the absence of Swd2.2. We explain how we have identified the genomic features that are common to the 61 genes either under- or over-expressed in the absence of Swd2.2.
(DOCX)
