# Supplementary figures and images for: CPF-Associated Phosphatase Activity Opposes Condensin-Mediated Chromosome Condensation
Source: PLoS Genet. 2014 Jun 19;10(6):e1004415. doi: 10.1371/journal.pgen.1004415 (PMC4063703; doi:10.1371/journal.pgen.1004415)

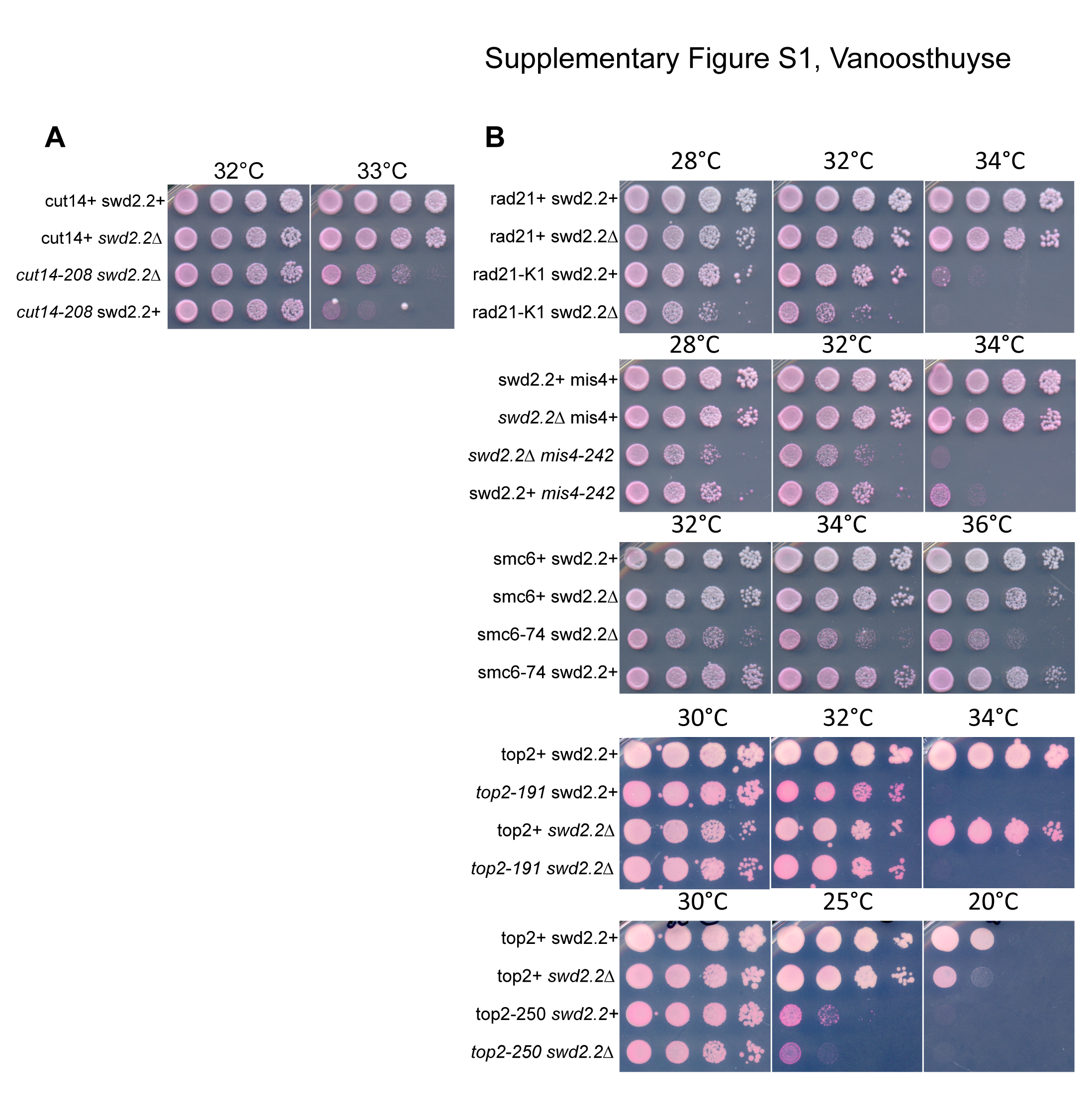

Supplement: Figure S1 — Lack of Swd2.2 does not improve the viability of other mutants defective in chromosome architecture. AB. Serial dilutions of the indicated strains were plated on rich media at the indicated temperatures. (TIF) [file pgen.1004415.s001.tif]

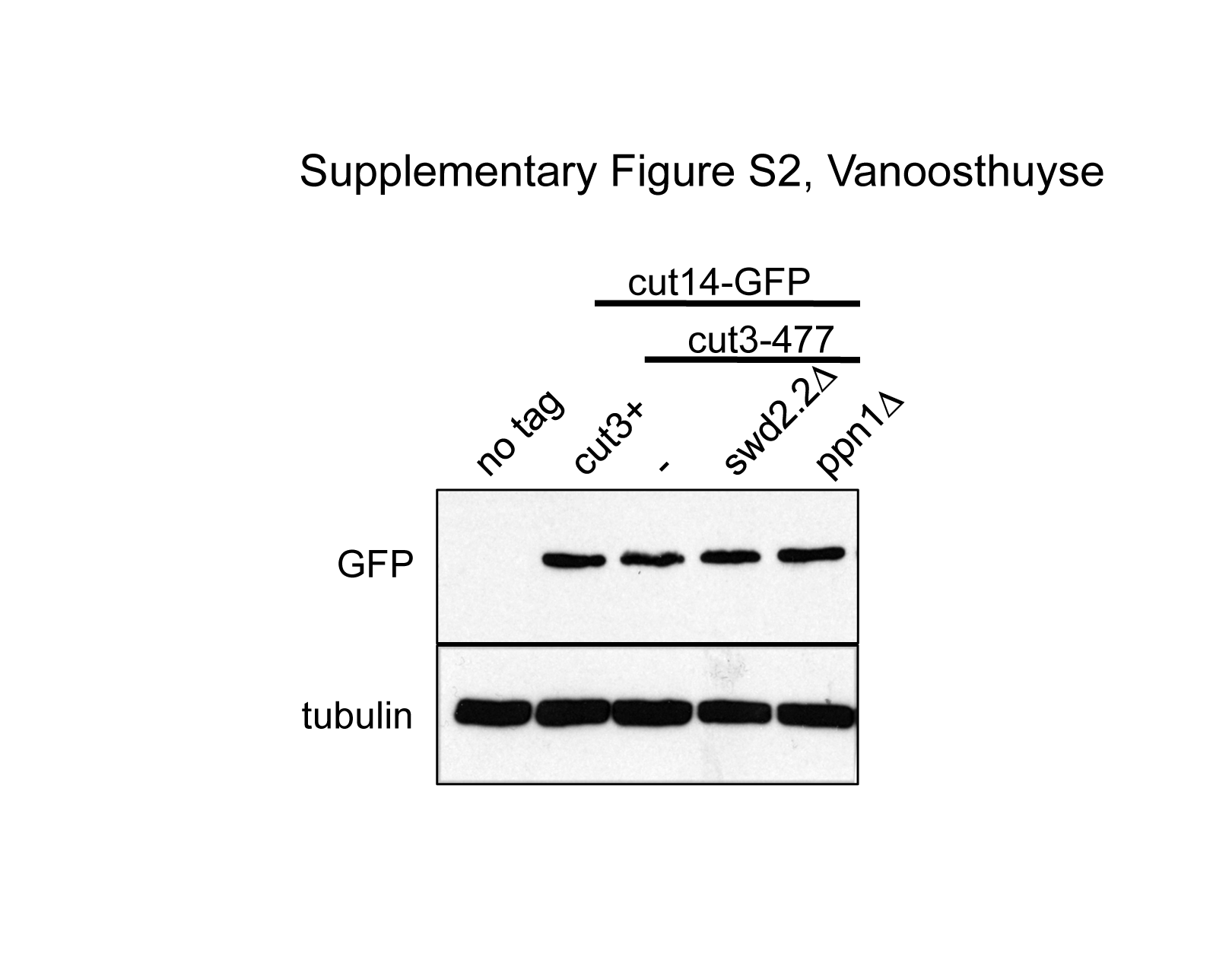

Supplement: Figure S2 — Lack of Swd2.2 does not alter the stability of Cut14 protein. Western blot analysis of the indicated strains. (TIF) [file pgen.1004415.s002.tif]

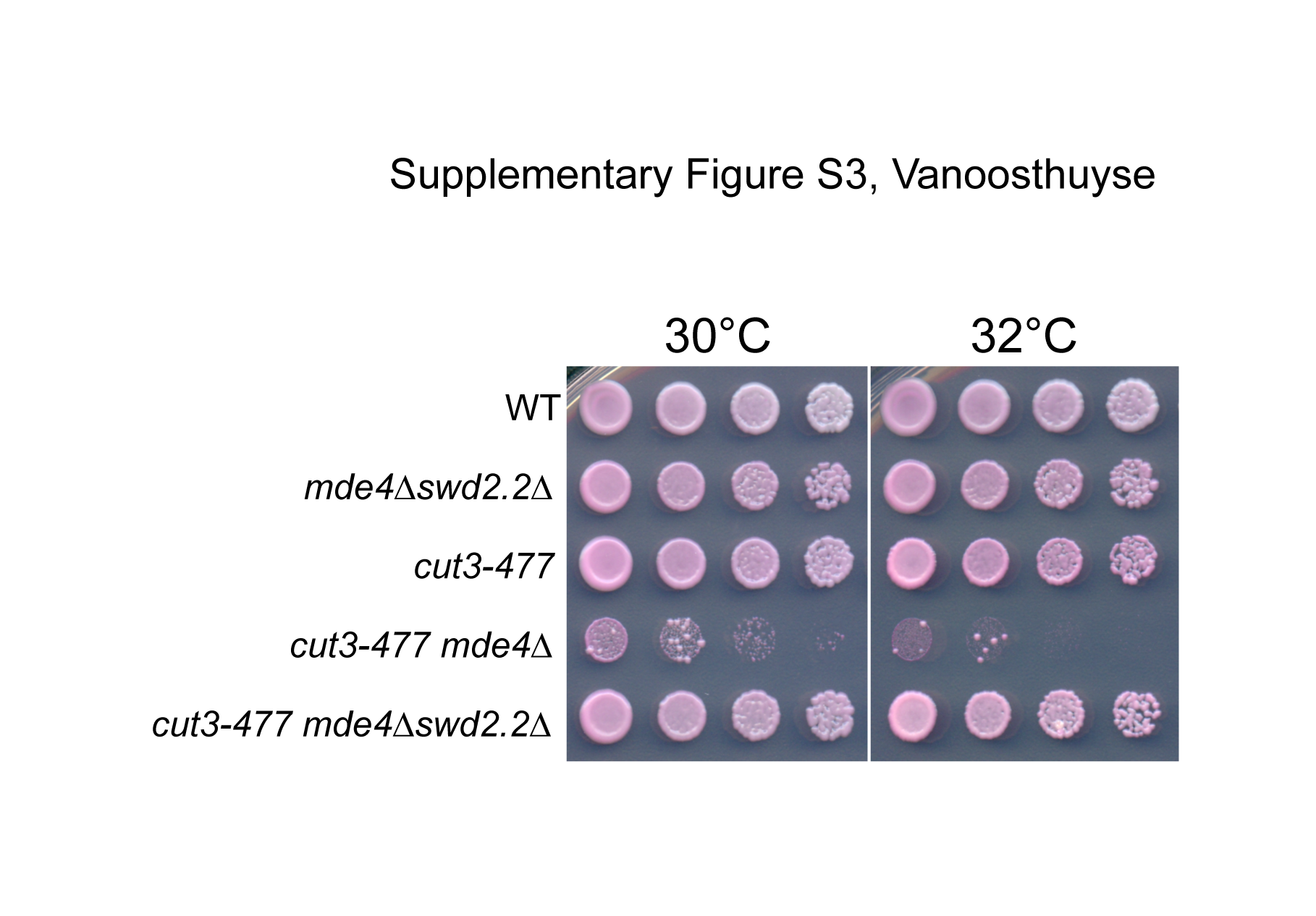

Supplement: Figure S3 — Lack of Swd2.2 suppresses the synthetic lethal interaction between cut3-477 and mde4Δ. Serial dilutions of the indicated strains were plated on rich media at the indicated temperatures. (TIF) [file pgen.1004415.s003.tif]

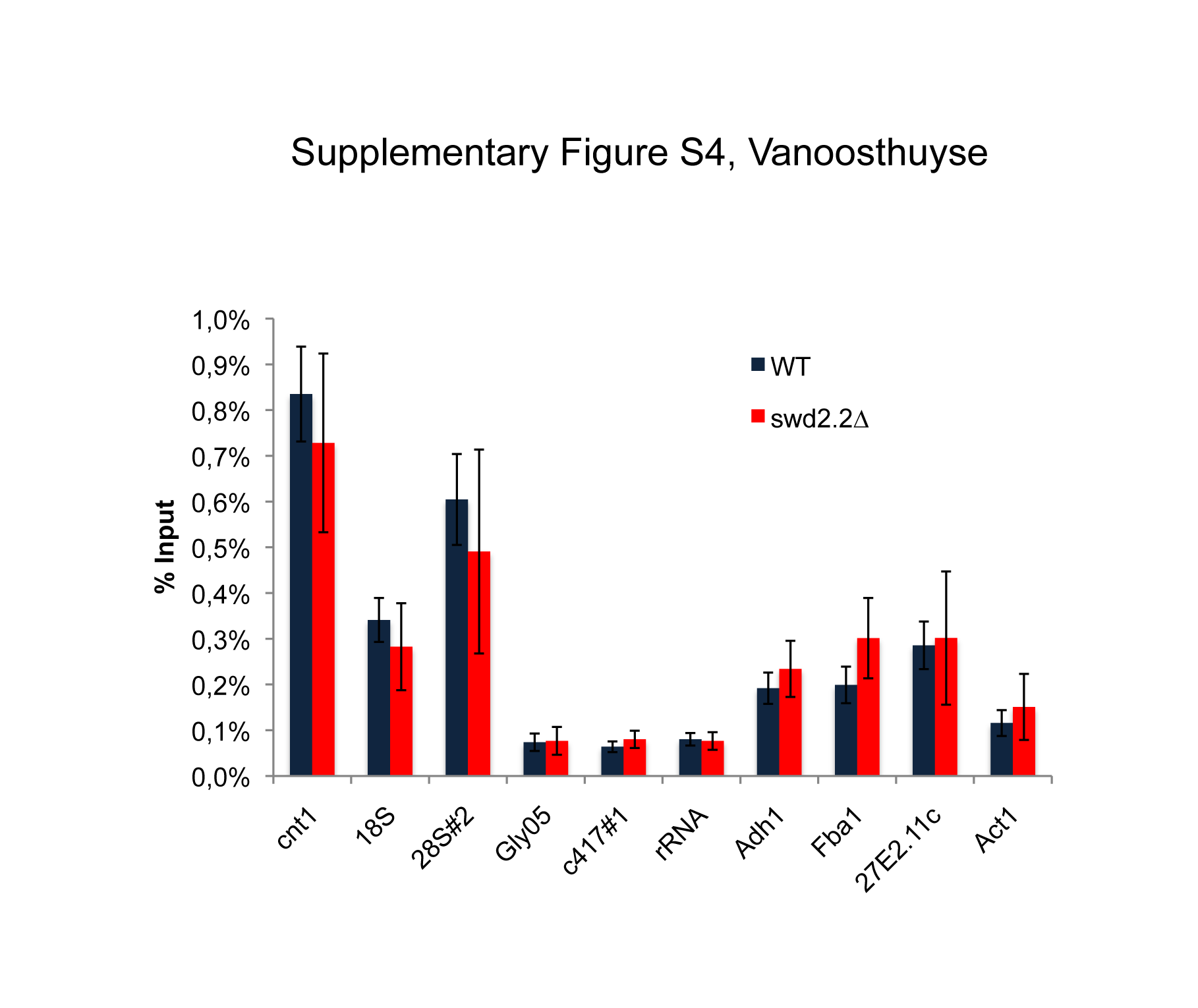

Supplement: Figure S4 — Lack of Swd2.2 does not significantly alter the localization of the wild-type condensin complex. ChIP qPCR of the GFP-tagged condensin sub-unit Cut3 (Cut3-GFP) in the presence or absence of Swd2.2. Strains were grown in cycling conditions in rich medium at 30°C (mean ± standard deviation from 3 biological replicates). (TIF) [file pgen.1004415.s004.tif]

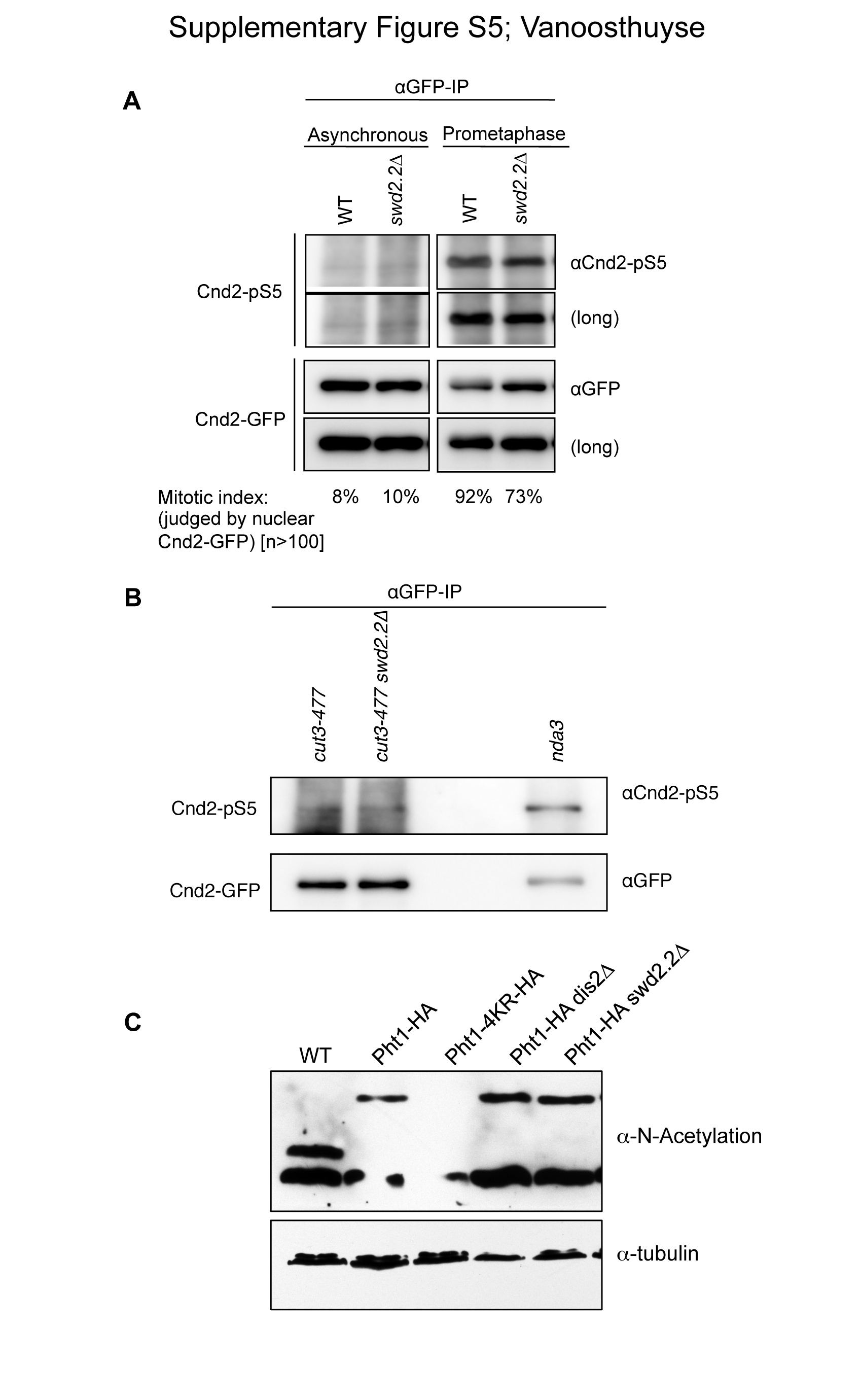

Supplement: Figure S5 — Lack of Swd2.2 has no detectable effect on Aurora B-dependent Cnd2- phosphorylation or H2A.zPht1 acetylation. A. Cycling or early mitotic cells (arrested in prometaphase with the cold-sensitive nda3-KM311 mutation) expressing the condensin sub-unit Cnd2 tagged with GFP at its endogenous locus were imaged under the microscope to count the number of mitotic, GFP-positive cells. Protein extracts were prepared from the same cells and GFP-tagged Cnd2 was immuno-precipitated. The immuno-precipitated complexes were analyzed by western blot using an antibody recognising the phospho-modified form of Cnd2, as described previously [2]. The mitotic indexes in each population is indicated underneath, based on the number of GFP-positive cells in the population. B. The same experiment as in (A) was repeated with cut3-477 or cut3-477 swd2.2Δ cells expressing GFP-tagged Cnd2 grown at the restrictive temperature of 34°C for one generation (3 hours). As the mitotic index is much smaller in these cells compared to nda3KM311-arrested cells, a diluted “nda3” samples was put on the same gel as control. C. Lack of Swd2.2 has no detectable effect on the acetylation of H2A.zPht1. Protein extracts were prepared from cells expressing HA-tagged Pht1 and analyzed by western blot using an acetyl-specific antibody recognising the acetylated isoform of Pht1 (see [22]). A HA-tagged, non-acetylable version of Pht1 (Pht1-4KR) was used as a control. (TIF) [file pgen.1004415.s005.tif]

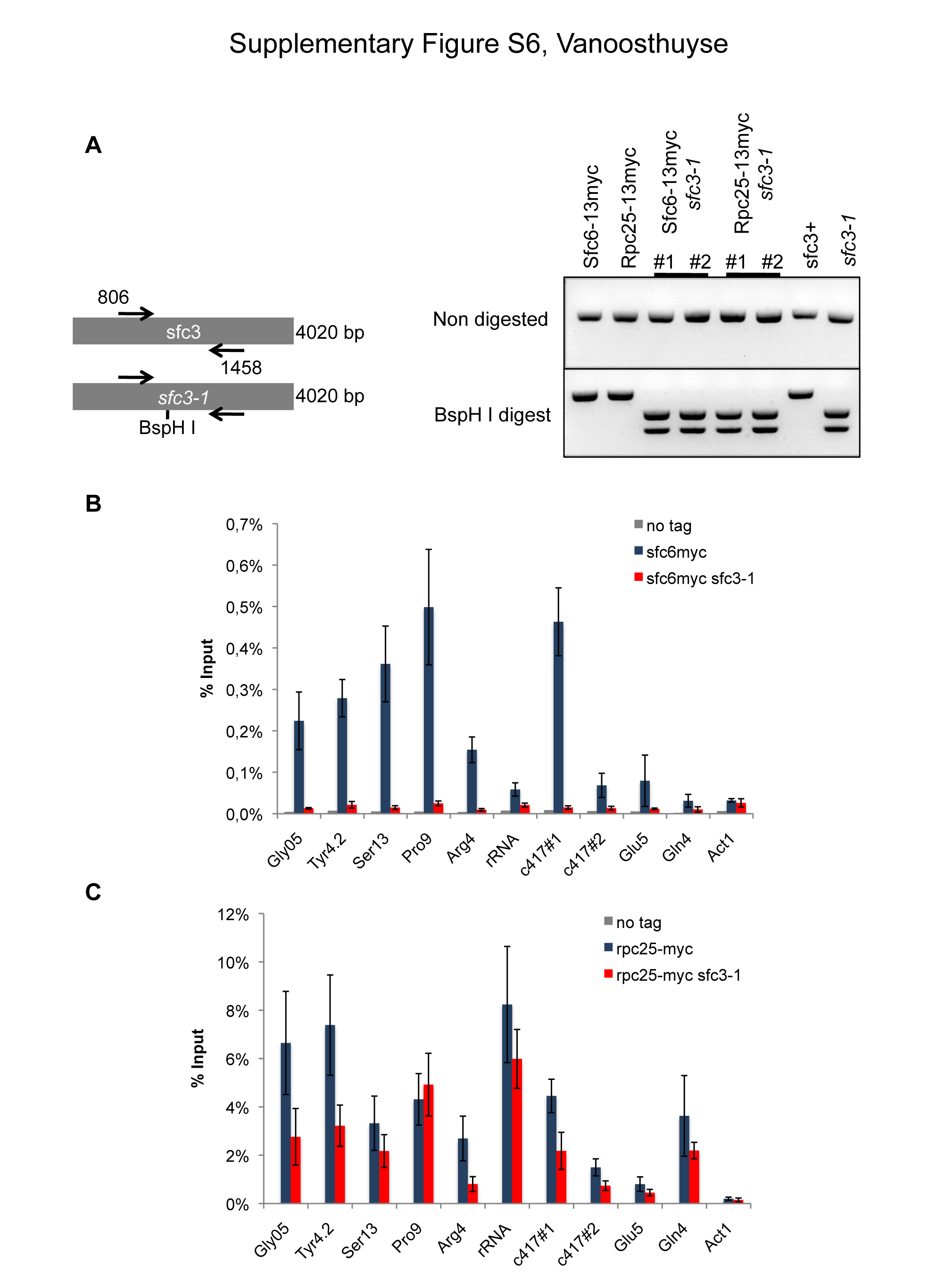

Supplement: Figure S6 — The association of Sfc6 and Rpc25 at Pol III-transcribed genes is impaired in sfc3-1 cells. A. Genotyping of the sfc3-1 mutation. (left panel) Method: the sfc3-1 mutation creates a BspH I site. For genotyping, a PCR product (fragment 806–1458 bp) is digested with BspH I for 2 hours. PCR products derived from sfc3+ cells remain undigested (right panel). Genotyping of the strains used for the ChIP in B and C. BC. ChIP qPCR of the indicated strains grown in cycling conditions for 2 hours at 36°C, the restrictive temperature of sfc3-1 (mean ± standard deviation from 6 biological replicates). (TIF) [file pgen.1004415.s006.tif]

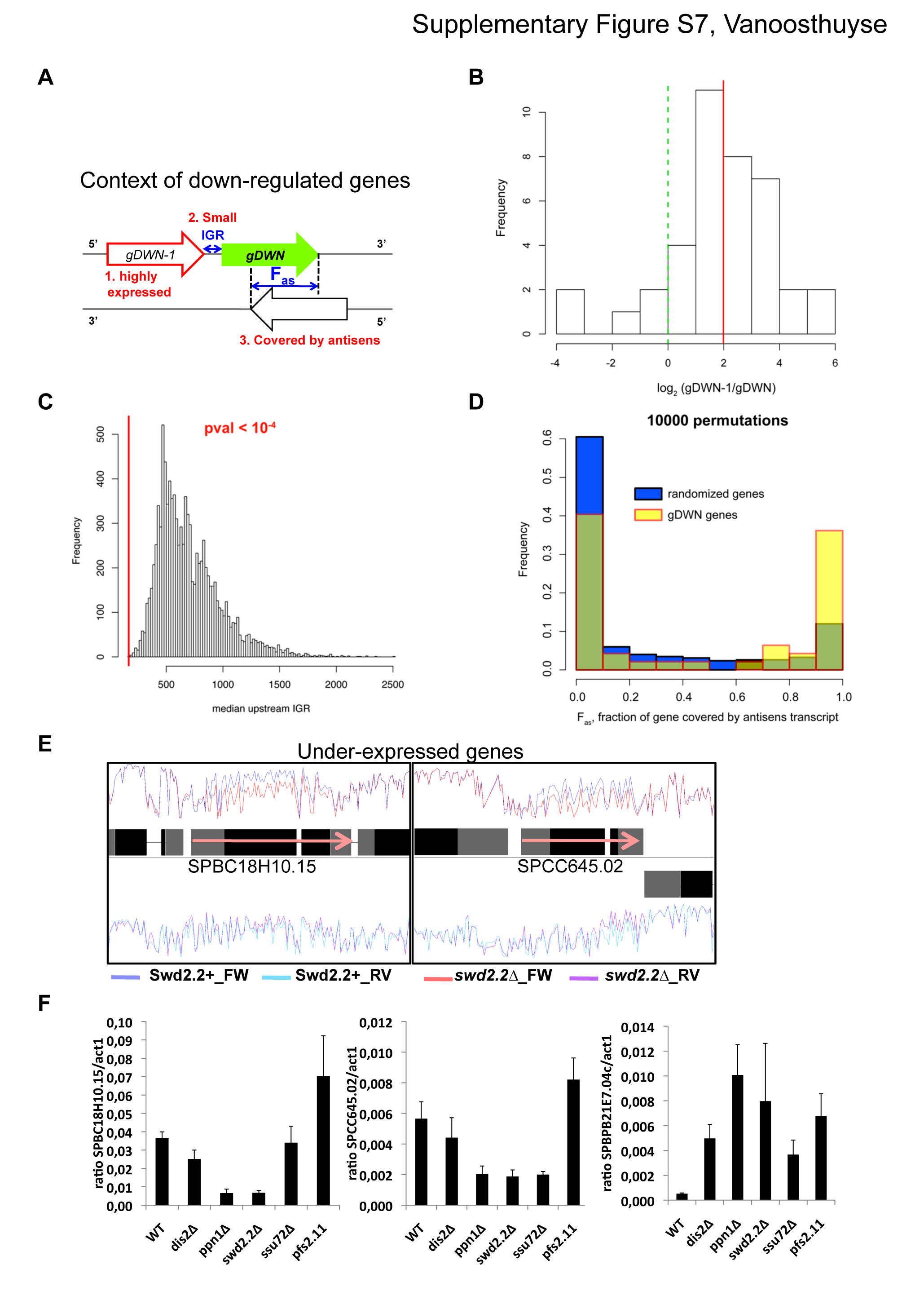

Supplement: Figure S7 — Genomic context of the 47 down-regulated genes in cells lacking Swd2.2. A. Scheme explaining the genomic context of the 47 down-regulated genes. gDWN = gene of interest; gDWN-1 = gene positioned on the same DNA strand directly upstream of g. IGR: Intergenic distance in bp between the end of the 3′UTR of gDWN-1 and the beginning of the 5′UTR of gDWN. Fas Fraction of gDWN covered by an antisense transcript. B. Distribution of expression differences between gDWN and gDWN-1 in the wild-type context. X-axis: log2 ratio of the expression level of gDWN-1 over the expression level of gDWN. Y-axis: gene counts. Sums of all bars = 47, representing all pairs (gDWN-1; gDWN) for the 47 gDWN genes under-expressed when Swd2.2 is absent. Red vertical line: median value. Green dotted line indicates the expected median if gDWN-1 and gDWN had similar expression levels. C. The median IGR observed for the 47 gDWN genes (red line) is shorter than for other genes of the genome. 10,000 sets of 47 genes were drawn at random from the genome, and the median IGR computed for each set. The black histogram shows the distribution of the resulting 10,000 median values. All of them exceeded the value observed on the actual set of 47 gDWN genes. D. gDWN genes are more often covered by an antisense transcript than other genes of the genome. X-axis: fraction of gene covered by antisense transcription (Fas). The yellow histogram shows the distribution of Fas values for the 47 gDWN genes. For comparison, the blue histogram shows the distribution of Fas values calculated for 10,000 random sets of 47 genes picked in the genome. The two distributions were significantly different (Kolmogorov-Smirnov P-value = 0.0004). This diagram indicates that the majority of genes in the S.pombe genome are not covered by an antisense transcript (Fas = 0 for ∼60% of genes, blue histogram), whilst roughly 10% of genes are completely covered by an antisense transcript (Fas = 1, blue histogram). For the 47 gDWN genes, the pe [file pgen.1004415.s007.tif]

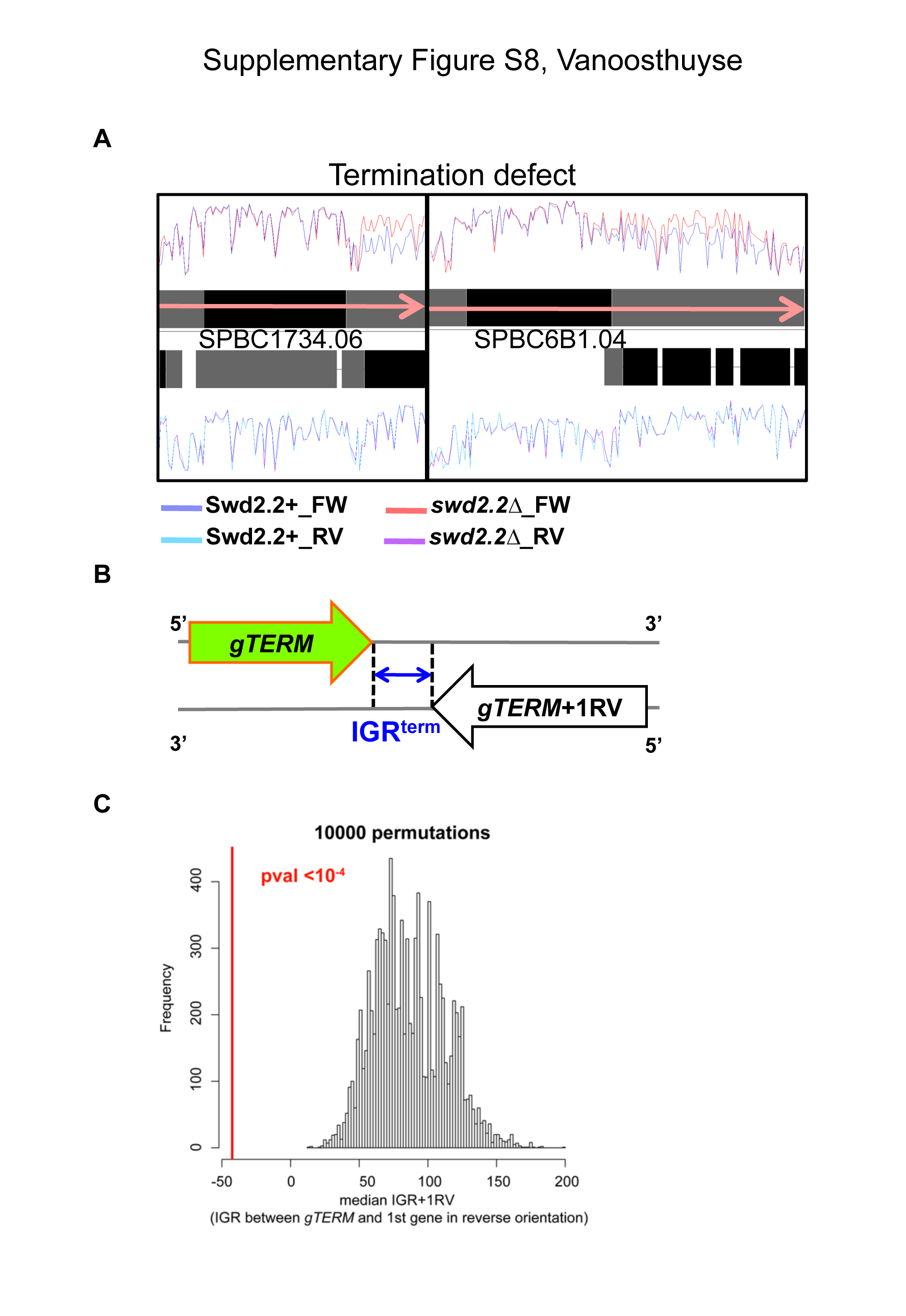

Supplement: Figure S8 — The effect of Swd2.2 on transcription termination is context-dependent. A. Tiling-array hybridization intensity profiles along two genes showing transcription termination defects when Swd2.2 is missing. Black boxes correspond to the coding region of the genes and grey boxes correspond to the UTRs. White boxes correspond to introns. The arrow indicates the orientation of transcription. B. Scheme explaining the genomic context of the genes with transcription termination defects in the absence of Swd2.2. gTERM = gene of interest showing no difference of microarray signal in the coding region but a stronger signal in the 3′UTR when Swd2.2 is missing. gTERM+1RV: the first downstream gene in the reverse orientation. IGRterm: IGR regions located between gTERM and gTERM+1RV. C. The size of IGRterm was measured for each gene of interest. Red vertical line = median size measured for the 780 gTERM genes. It is negative, showing that most gTERM genes overlap with their immediate downstream reverse gene. Black histogram: distribution of median values obtained on 10,000 random sets of 780 genes. (TIF) [file pgen.1004415.s008.tif]

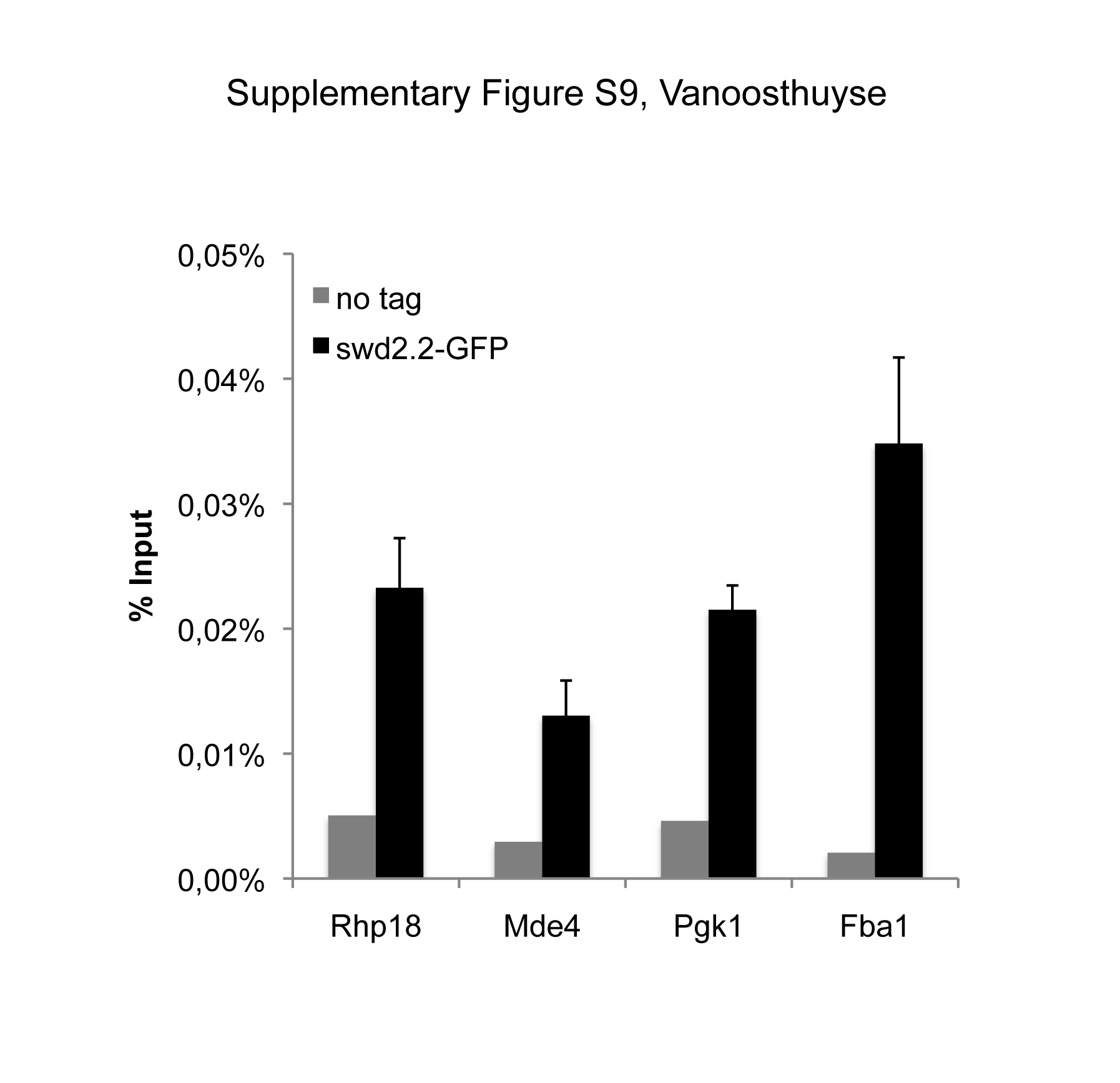

Supplement: Figure S9 — Enrichment of Swd2.2 established by ChIP at convergent genes exhibiting transcription termination defects when Swd2.2 is missing. Rhp18 and Mde4 are two genes placed in situation of convergence with respectively SPBC1734.07c and Atg7, whose transcription termination is affected in the absence of Swd2.2. Fba1 and Pgk1 suffer no transcription termination defect in the absence of Swd2.2. ChIP analysis showed that Swd2.2 is not more abundant in the 3′UTR of Rhp18 and Mde4 compared to the 3′UTR of Fba1 and Pgk1 (mean ± standard deviation from 5 biological replicates). (TIF) [file pgen.1004415.s009.tif]

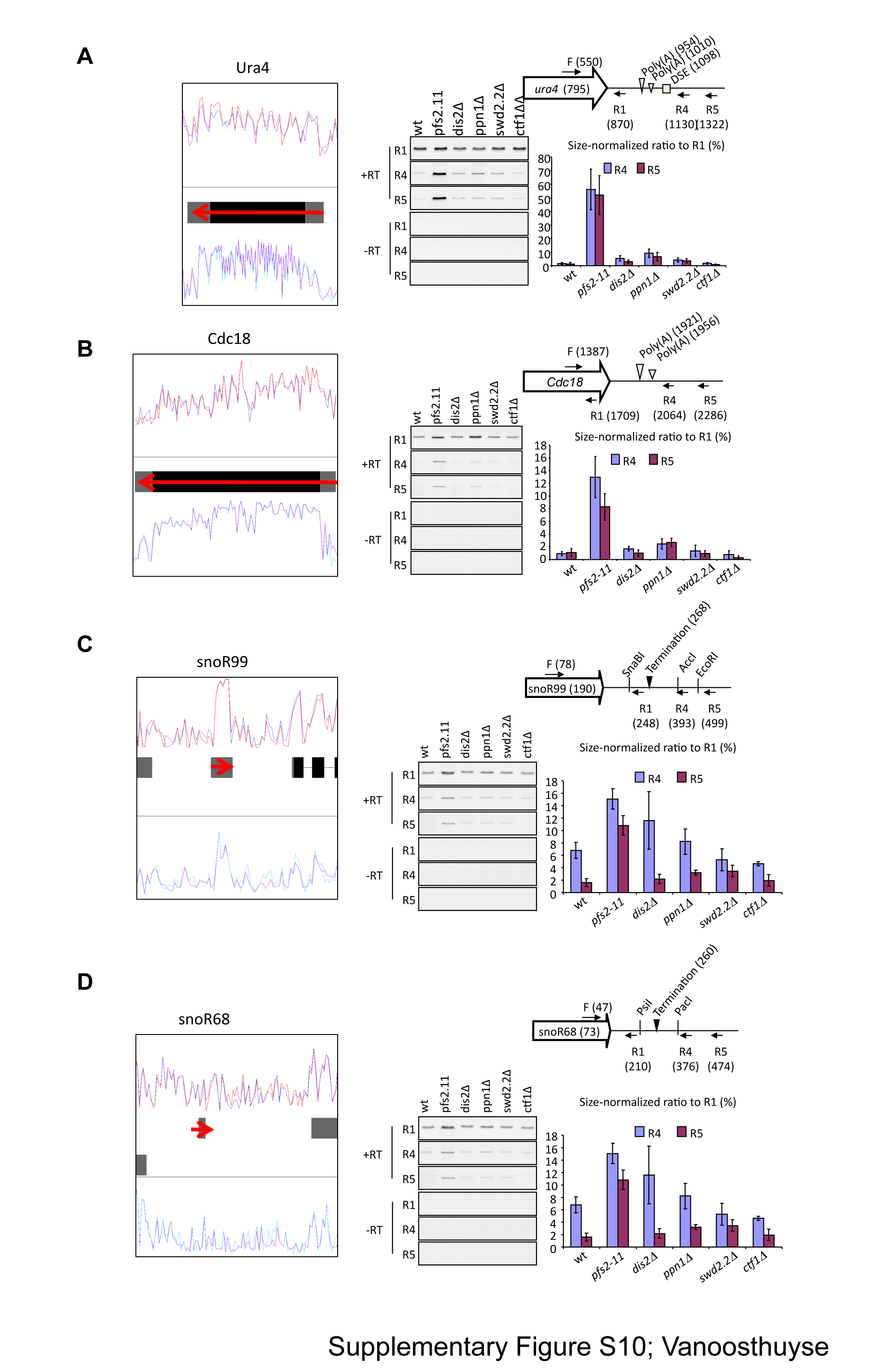

Supplement: Figure S10 — Lack of DPS has a moderate effect on transcription termination at snoRNAs. Transcription termination was monitored in deletion mutants of various CPF-associated proteins at Ura4+ (A), Cdc18 (B), snoR99 (C) and snoR68 (D). Each panel follows the same organization: on the left, the tiling-array hybridization intensity profiles along the gene of interest is displayed for each strand as seen for Swd2.2+ or swd2.2Δ cells; on the right, the result of a qRT-PCR strategy designed to quantify the proportion of RNA that have been transcribed passed the previously-identified site of transcription termination. Termination sites are identified on the figure together with genomic features such as restriction sites. For each gene examined, the PCR product F-R1 served as internal loading control. The histograms show the size-normalized ratio to R1 = (band intensity/band size)/(R1 band intensity/R1 size). For each, n = 3 biological replicates. (TIF) [file pgen.1004415.s010.tif]

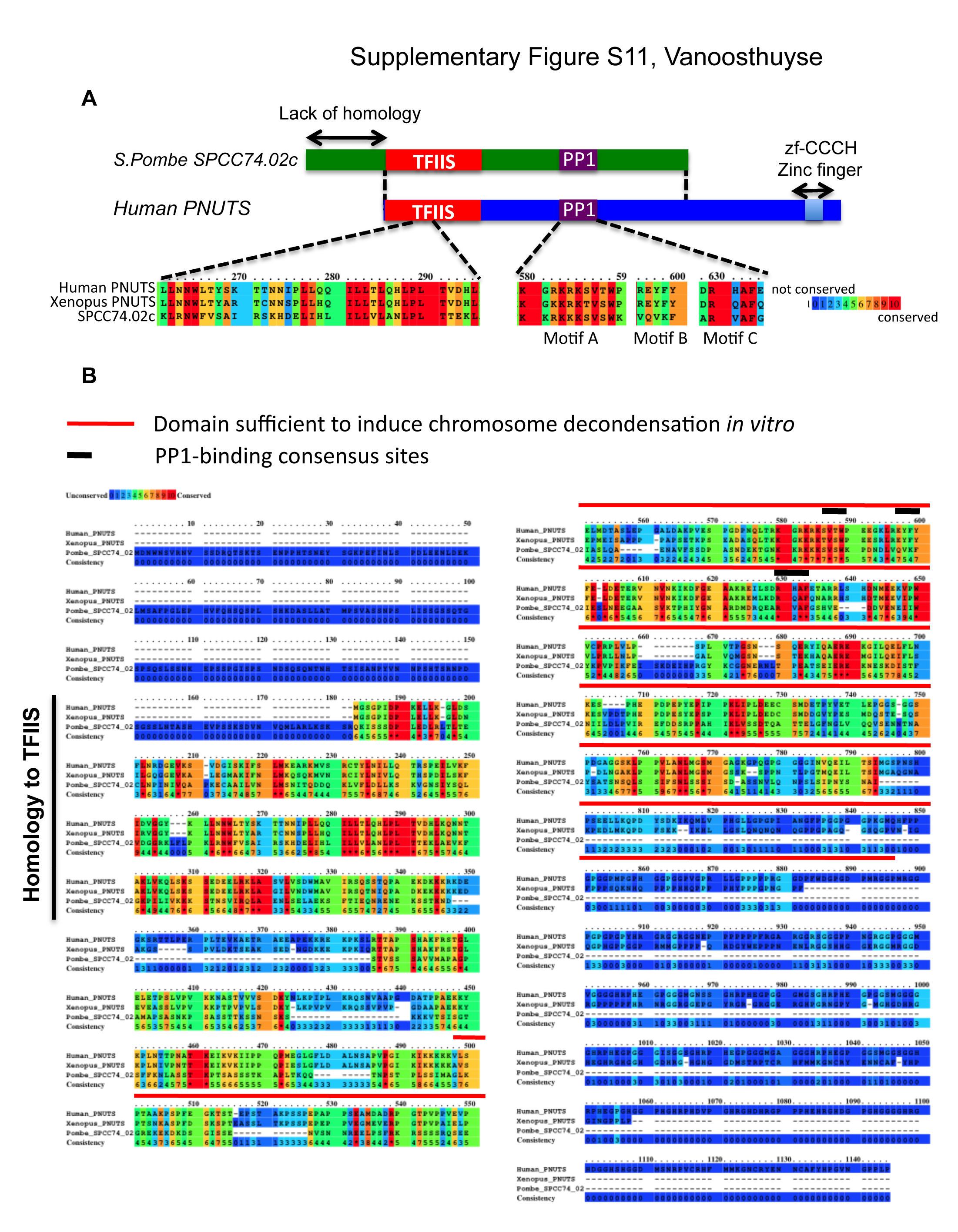

Supplement: Figure S11 — Ppn1 shows significant homology with human and xenopus PNUTS. A. Scheme explaining the domain organization of the PNUTS homologues in Human and S.pombe. The domain showing homology to TFIIS, and the PP1-binding consensus sites are highlighted. B. The PRALINE software (http://www.ibi.vu.nl/programs/pralinewww/) was used to create an alignment between the human and Xenopus PNUTS homologues and the fission yeast SPCC74.02c (Ppn1). The region of human PNUTS found to be sufficient to induce chromosome decondensation in vitro [37] is underlined in red. The PP1-binding motifs are underlined in black. (TIF) [file pgen.1004415.s011.tif]

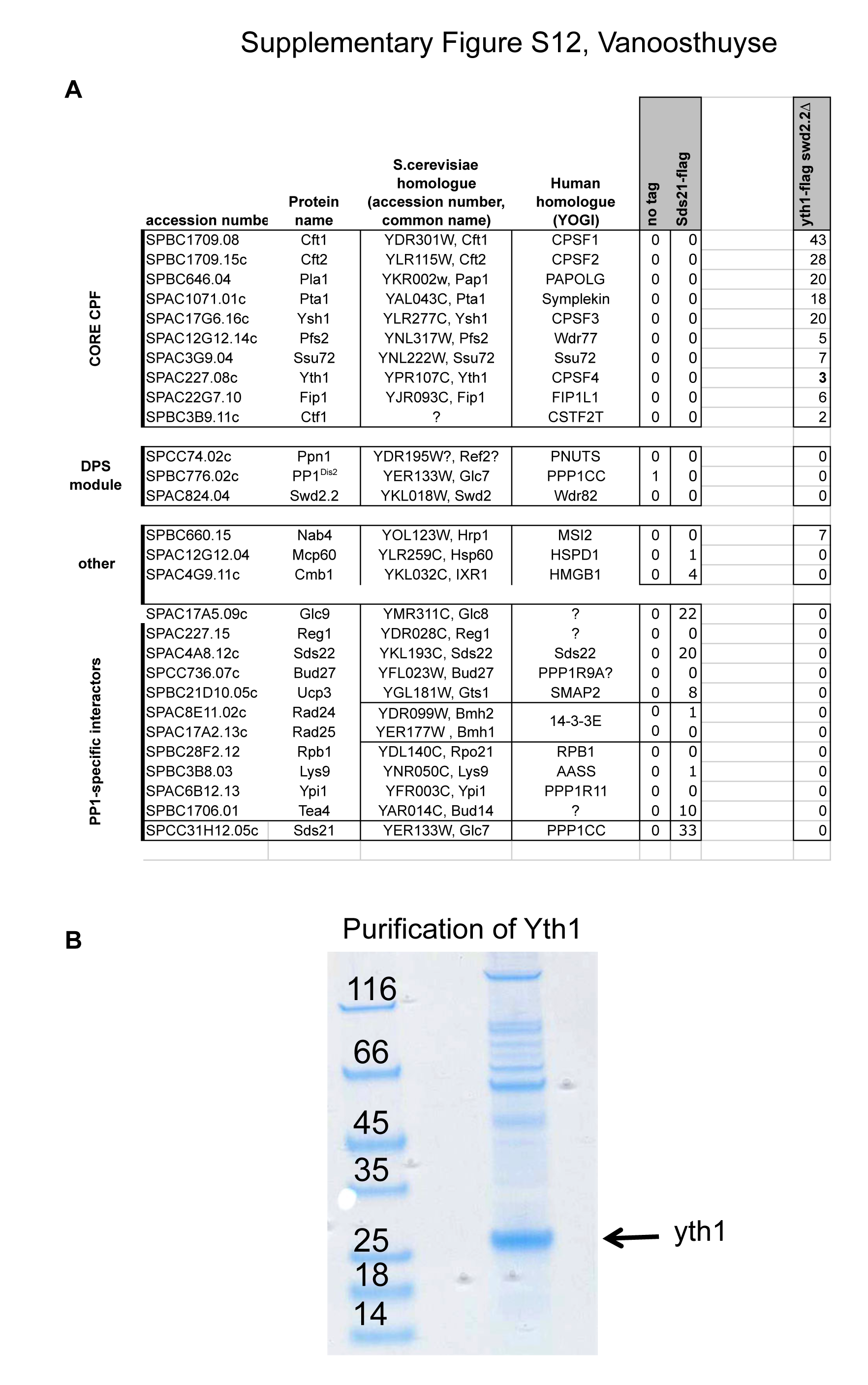

Supplement: Figure S12 — Mass-spectrometry analysis of Sds21- and Yth1-associated proteins. A. The proteins indicated at the top were affinity purified and the associated proteins identified by MS/MS mass-spectrometry analysis (see Methods). The number of unique peptides recovered for each protein is indicated. B. The proteins recovered after affinity purification of Flag-tagged Yth1 were run on an SDS-PAGE gel and visualized by coomassie staining. (TIF) [file pgen.1004415.s012.tif]

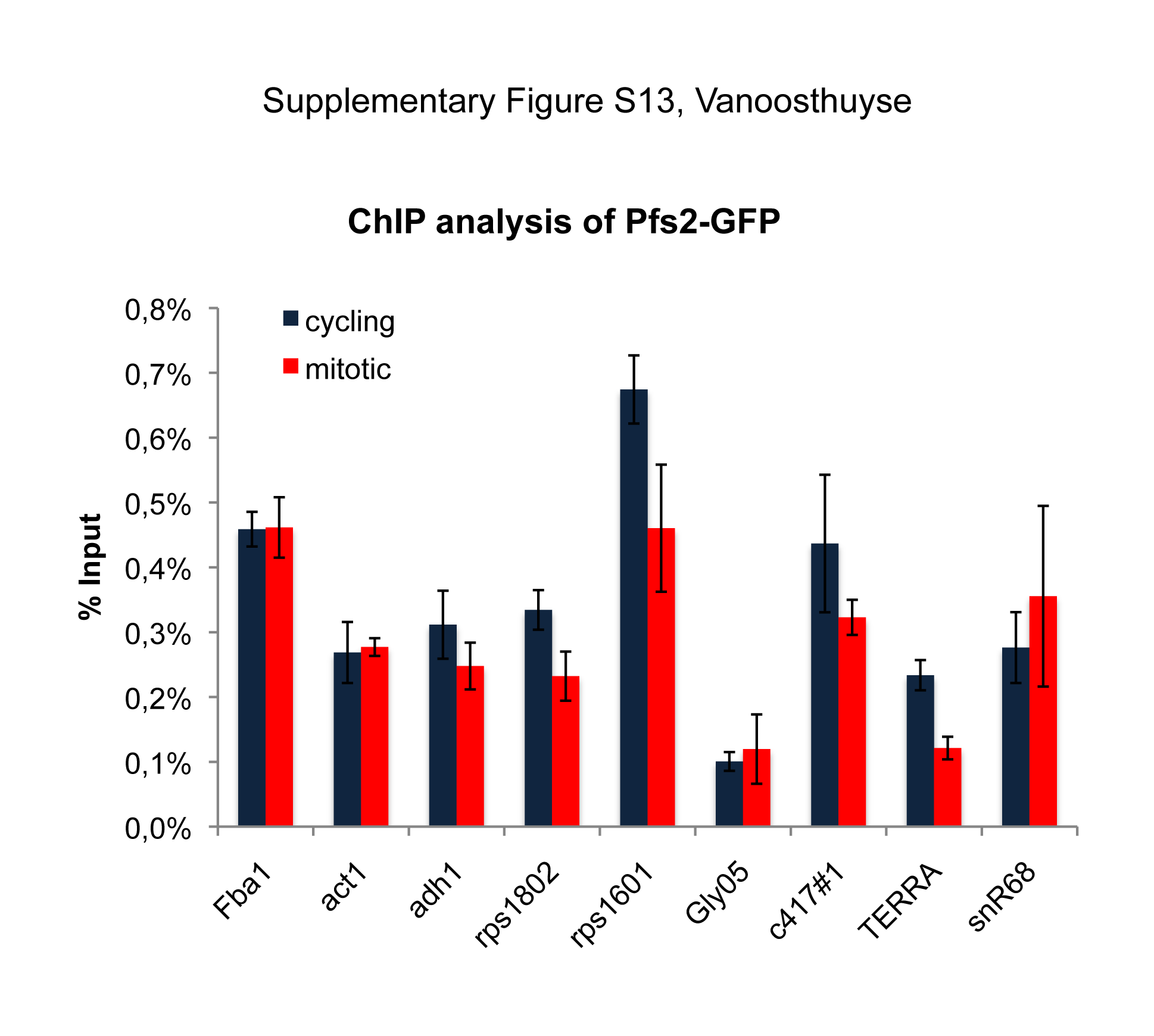

Supplement: Figure S13 — The CPF sub-unit Pfs2 remains associated with chromatin in early mitotic cells. ChIP-qPCR analysis was performed to monitor the association of GFP-tagged Pfs2 with chromatin in cycling cells and in cells synchronized at the metaphase to anaphase transition using the cold-sensitive tubulin mutation nda3KM311 (mean ± standard deviation from 6 biological replicates). (TIF) [file pgen.1004415.s013.tif]

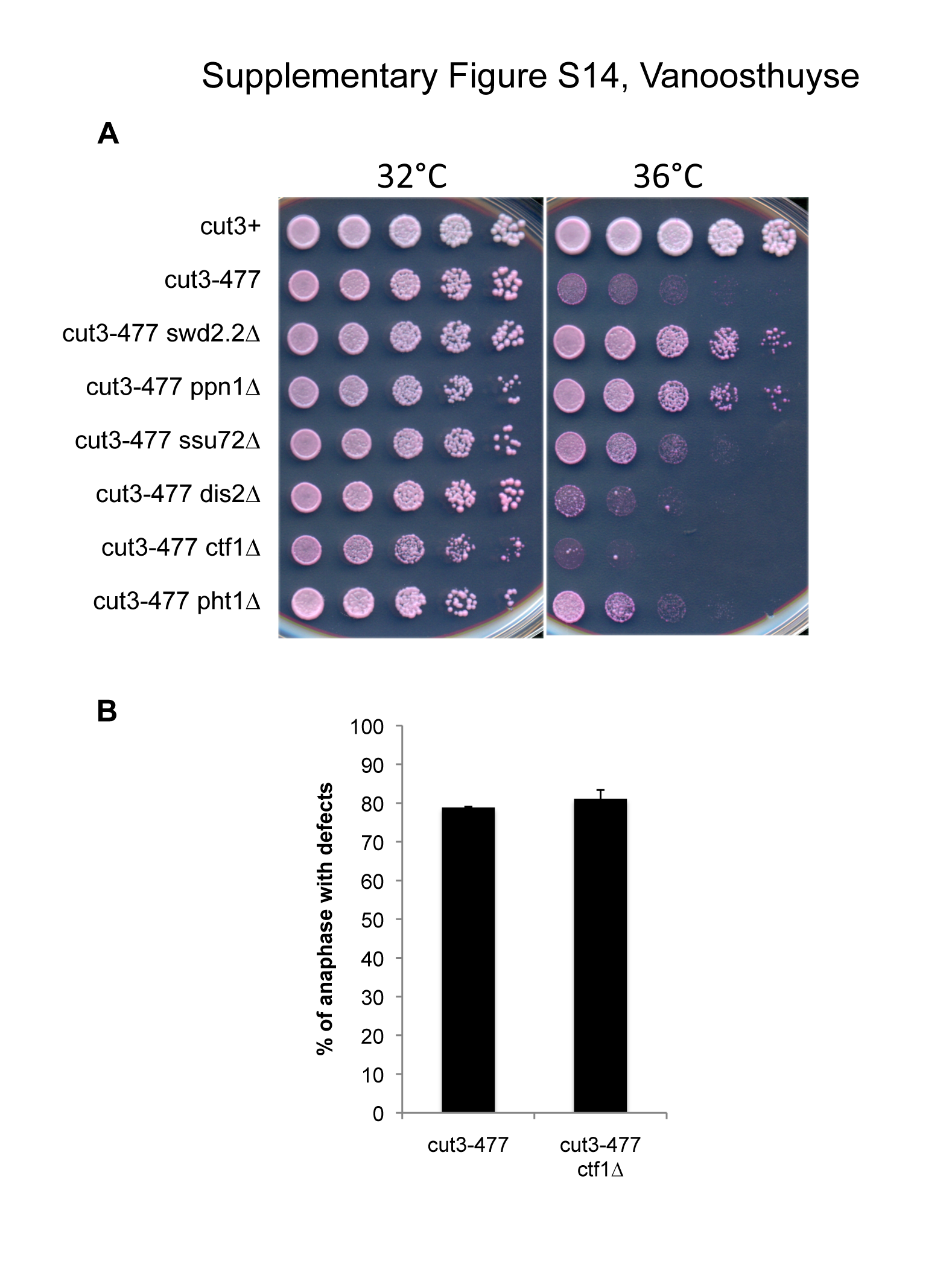

Supplement: Figure S14 — The CPF component Ctf1 does not oppose condensin-mediated chromosome condensation. A. Serial dilutions of the indicated strains were plated on rich media at the indicated temperatures. B. Chromosome segregation in anaphase was visualized after growing cells of the indicated genotypes for one generation at 34°C. Anaphases were scored as defective when lagging chromatin was detected between the two main DNA masses. For each genotype, 3 independent experiments were performed in which a minimum of 100 anaphase cells was scored. (TIF) [file pgen.1004415.s014.tif]

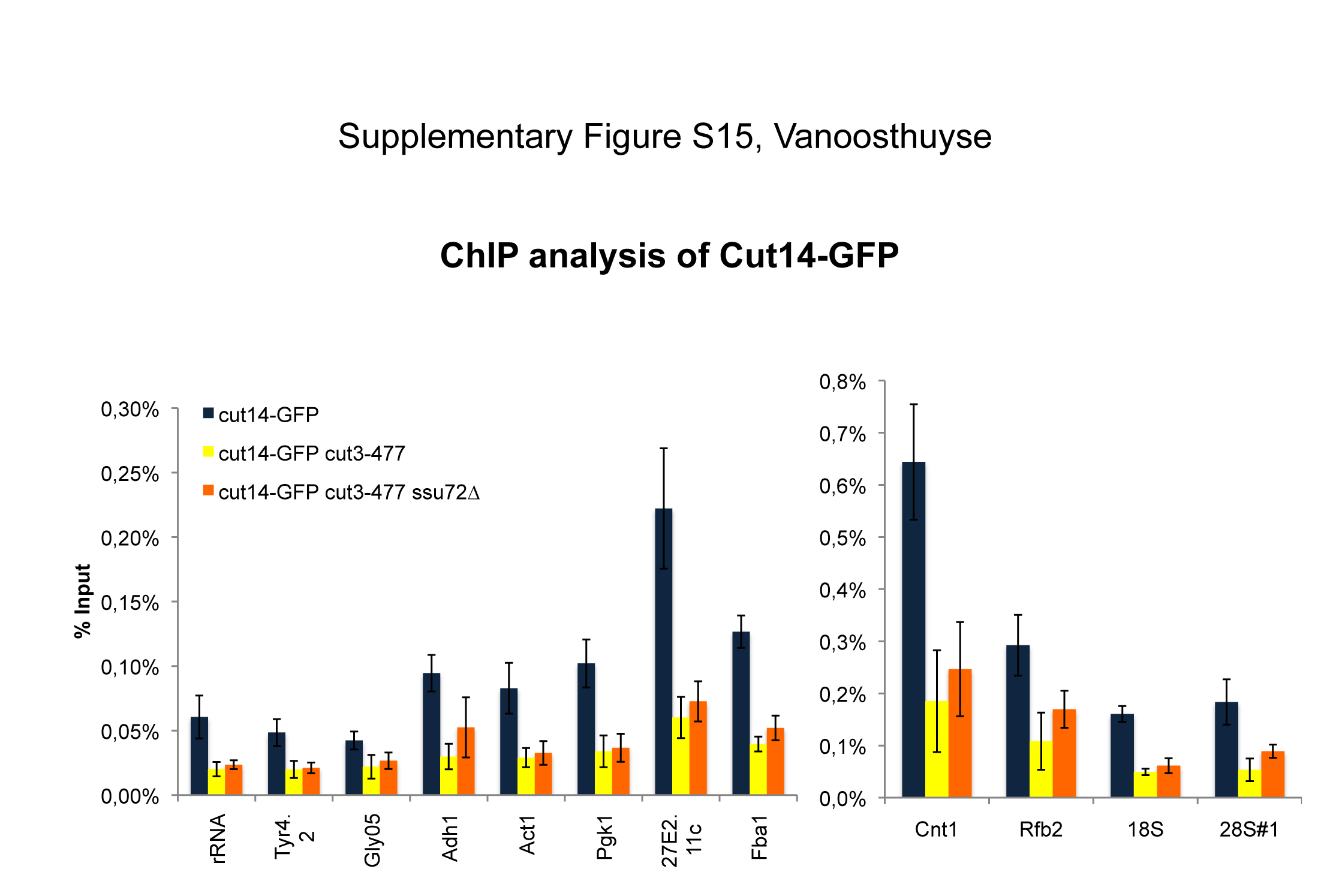

Supplement: Figure S15 — Lack of Ssu72 does not restore the localization of condensin in cut3-477 cells. The indicated strains were grown at 34°C for 3 hours and ChIP-qPCR was performed to analyze the amount of Cut14-GFP cross-linked to chromatin (mean ± standard deviation from 6 biological replicates). The enrichments of Cut14 observed in cut3-477 and cut3-477 ssu72Δ cells were not significantly different (p>0,2, Wilcoxon - Mann Whitney). (TIF) [file pgen.1004415.s015.tif]
