# Supplementary material for: CPF-Associated Phosphatase Activity Opposes Condensin-Mediated Chromosome Condensation
Source: PLoS Genet. 2014 Jun 19;10(6):e1004415. doi: 10.1371/journal.pgen.1004415 (PMC4063703; doi:10.1371/journal.pgen.1004415)
Supplement: Text S1 — Analysis of the genomic context surrounding the genes that are mis-expressed in the absence of Swd2.2. We explain how we have identified the genomic features that are common to the 61 genes either under- or over-expressed in the absence of Swd2.2. (DOCX) [file pgen.1004415.s021.docx]

# Text S1: Analysis of the genomic context surrounding the genes mis-expressed when Swd2.2 is missing

We asked whether the 61 genes whose expression is altered in the absence of Swd2.2 share common features. In all cases but two, the expression of the neighbouring genes is not significantly affected (data not shown). Both under- (thereafter called *gDWN*) and over-expressed (thereafter called *gUP*) genes correspond to genes moderately expressed in Swd2.2+ cells (data not shown). Remarkably, the genomic environments surrounding *gDWN* and *gUP* genes are radically different. *gDWN* genes exhibit three specific features (Figure S7): (1) the gene placed directly upstream of *gDWN* on the same DNA strand (called thereafter *gDWN-1*) is expressed to significantly higher levels than *gDWN* in Swd2.2+ cells (Figure S7B). This difference was pronounced, with a median of 4-fold, and was statistically significant (*P* = 3.4x10^-6^, see methods); (2) the intergenic distance between *gDWN*-1 and *gDWN* is statistically very small, with a median of 115 bp. Such a small median distance was never observed among 10,000 random sets of genes (Figure S7C). On the contrary, *gUP* genes tend to be isolated: in this case, the median intergenic distance is 1393 bp. Finally, (3) *gDWN* genes are largely covered by antisense transcription units (Figure S7D). These observations highlight the specificity of the genomic context surrounding *gDWN* genes.

Given the very specific genomic context of *gDWN* genes, we first hypothesized that their downregulation in *swd2.2∆* could result from a failure to terminate transcription at *gDWN-1*. Indeed, if a gene is highly expressed (such as *gDWN-1* genes), failure to terminate transcription properly may have important consequences for the downstream transcription unit. We tested this hypothesis in two ways. First, using our tiling arrays dataset, we examined the expression profiles in swd2.2+ and *swd2.2∆* cells at the junction between *gDWN-1* and *gDWN*. We observed no significant differences between *swd2.2∆* and swd2.2+ cells, suggesting that transcription termination at *gDWN-1* is normal in the absence of Swd2.2 (Figure S7E). Then, using quantitative RT-PCR, we compared the expression of *gDWN* candidates in *swd2.2∆* and *pfs2-11* mutants. *pfs2-11* is a temperature-sensitive allele of the core CPF sub-unit Pfs2 with well-established transcription termination defects [1]. Strikingly, we observed that genes under-expressed in *swd2.2∆* and *ppn1∆* were in fact over-expressed in *pfs2-11*, suggesting that a transcription termination defect of *gDWN-1* results in the over-expression rather than the under-expression of *gDWN* (Figure S7F). Taken together, these observations are not consistent with the idea that a failure to terminate transcription at *gDWN-1* explains the reduced expression of *gDWN*.

**Supplementary Reference**

1. Wang SW, Asakawa K, Win TZ, Toda T, Norbury CJ (2005) Inactivation of the pre-mRNA cleavage and polyadenylation factor Pfs2 in fission yeast causes lethal cell cycle defects. Mol Cell Biol 25: 2288-2296.
